# Multi-class chemical exposure in rural Peru using silicone wristbands

**DOI:** 10.1038/jes.2017.12

**Published:** 2017-07-26

**Authors:** Alan J Bergmann, Paula E North, Luis Vasquez, Hernan Bello, Maria del Carmen Gastañaga Ruiz, Kim A Anderson

**Affiliations:** 1 Department of Environmental and Molecular Toxicology, Oregon State University, Corvallis, Oregon, USA; 2 Department of Pathology, Medical College of Wisconsin, Milwaukee, Wisconsin, USA; 3 Yantaló Peru Foundation, Yantaló, Moyobamba, Peru; 4 Centro Nacional de Salud Ocupacional y Protección del Ambiente para la Salud—CENSOPAS/INS, Lima, Peru

**Keywords:** biomonitoring, chemical mixtures, exposome, passive sampling device, Peru, South America

## Abstract

Exposure monitoring with personal silicone wristband samplers was demonstrated in Peru in four agriculture and urban communities where logistic and practical constraints hinder use of more traditional approaches. Wristbands and associated methods enabled quantitation of 63 pesticides and screening for 1397 chemicals including environmental contaminants and personal care products. Sixty-eight wristbands were worn for approximately one month by volunteers from four communities of Alto Mayo, Peru. We identified 106 chemicals from eight chemical classes among all wristbands. Agricultural communities were characterized by pesticides and PAHs, while the urban communities had more personal care products present. Multiple linear regressions explained up to 40% of variance in wristbands from chlorpyrifos, cypermethrin, and DDT and its metabolites (DDx) (*r*^2^=0.39, 0.30, 0.40, respectively). All three pesticides were significantly different between communities, and cypermethrin and DDx were associated with participant age. The calculated relative age of DDT suggested some communities had more recent exposure than others. This work aids health research in the Alto Mayo and beyond by identifying typical mixtures and potential sources of exposure to organic chemicals in the personal environment. Silicone wristband sampling with chemical screening is a candidate for widespread use in exposure monitoring in remote areas.

## Introduction

Exposure to exogenous chemicals is a substantial contributor to disease risk^1, 2^ and adverse effects may be exacerbated by other health states such as malnutrition.^3^ Many factors influence a person’s total exposure to toxicants^4^ which can result in highly individualized chemical environments.^2^ By characterizing the external personal environment, we can prioritize individual and mixtures of chemicals for toxicity and risk assessment. Techniques to measure personal exposure include internal and external tissue sampling,^4, 5^ personal air monitoring^6^ and hand wipes.^7^ Silicone wristbands were recently introduced as personal passive sampling devices (PSDs).^8^

PSDs are frequently used as stationary monitors of the environment.^9, 10, 11^ They operate by concentrating organic compounds over time, which improves environmental detection limits and captures episodic exposures. By sampling through passive diffusion into a hydrophobic polymer, such as low-density polyethylene (LDPE) or polydimethylsiloxane (silicone),^12^ many PSDs mimic uptake of chemicals by lipid tissue.^9^ Although silicone PSDs predominately sample lipophilic compounds, O’Connell et al. identified chemicals with logK_ow_s (log octanol-water partition coefficient) from −0.07 to 9.5 in extracts of silicone wristbands.^8^ Simply worn on the wrist, the silicone samples from any media it contacts. Therefore the wristbands capture chemicals from inhalation and dermal routes of exposure but do not account for ingestion. Recent work showed that concentrations of organophosphate flame retardants and polycyclic aromatic hydrocarbons (PAHs) in wristbands correlated with urinary metabolites of those compounds.^7, 13^ These studies support a link between the ambient personal environment sampled by the wristbands and the internal environment. As mixed-media samplers, the calculation of environmental concentrations is difficult, but wristbands can be used to evaluate relative exposures.^8, 14, 15^ PSDs are well suited for sampling in remote regions. A previous study showed that PAHs and pesticides are stable in low-density polyethylene PSDs transported in polytetrafluoroethylene (PTFE) bags for multiple weeks at ambient temperatures.^16^

The current study examined wristbands worn by volunteers in the Alto Mayo, a heavily deforested region with developing agriculture in the Department of San Martin in Northeast Peru. Much of Latin America is increasingly urbanized and industrially developed, exposing residents to more anthropogenic chemicals, while still experiencing hazards of infection by tropical diseases and malnutrition.^1^ Land use intensification of low-middle income countries is associated with pesticide use,^17^ but little information is available about the Alto Mayo. A series of reports surveyed pesticide application, environmental distribution, and blood concentrations of farm workers from Tarapoto, located 80 km southeast of the Alto Mayo.^18, 19, 20^ The compounds detected in blood and surface water near Tarapoto included p,p′-dichlorodiphenyldichloroethylene (DDE),^18, 19^ a transformation product of DDT banned in Peru in accordance with the Stockholm Convention.^19, 21^ Other legacy and contemporary use pesticides in Tarapoto indicated a mixture of historical and current sources of exposure.^18, 19^ They described farm workers who mixed and applied pesticides with no personal protection equipment, storage of pesticides in residences, and that children would sometimes help in agriculture fields.^20^ These practices have been documented elsewhere^22, 23^ and could distribute pesticide exposure among community members.

Here, we determined the distribution of pesticides and many other chemicals in the personal environment of Alto Mayo residents using silicone wristbands. We hypothesized that wristbands would accumulate different suites of chemicals in different communities, providing evidence for differential exposure based on lifestyle. We anticipated that pesticides would be greater in wristbands worn by farm workers and that in non-farming communities we would observe more urban signature such as elevated personal care products. By identifying typical mixtures of exposure, we provided critical preliminary data for health research in the Alto Mayo and demonstrated the applications and limitations of silicone wristbands as monitors of the personal environment.

## Materials and methods

### Materials

All solvents were purchased from Fisher Scientific (Pittsburgh, PA, USA). Methanol, isopropanol, dimethyl sulfoxide (DMSO), and hydrochloric acid (HCl) were ACS grade. Ethyl acetate was Optima and n-hexane was GC-Resolv. Standards were purchased at >99% purity. Tetrachloro-*m*-xylene (TCMX) was from Supelco (Bellefonte, PA, USA), polychlorinated biphenyl (PCB)-100, PCB-209, and *p,p’-*dibromooctafluorobiphenyl were from Accustandard (New Haven, CN, USA), and PCB-65-d5, PCB-115-d3, and PCB-156-d3 were from C/D/N Isotopes (Pointe-Claire, QC, Canada). Water was filtered through an in-house 18 MΩ*cm purification system (Millipore, Merck, Darmstadt, Germany).

### Environmental Passive Sampling

To get an overview of what pesticides and other compounds might be present, we conducted an initial survey of bioavailable contaminants in surface water of the Alto Mayo in August 2013. Methods followed previous deployment and analysis of LDPE passive sampling^24, 25^ and are described in detail in the supporting information.

### Personal Passive Sampling

Silicone wristbands were prepared as described previously.^8^ Briefly, wristbands were cleaned with three rounds of soaking in 1:1 ethyl acetate:n-hexane, then two rounds in 1:1 ethyl acetate:methanol. This cleaning was shown to remove the majority of background compounds, mainly siloxanes, from wristbands.^8^ Cleaned wristbands were vacuum-dried and transferred to individually sealed PTFE bags for transport to Peru. Two sizes of wristbands were used and were weighed before solvent cleaning. Large and small wristbands were 5.68 g (SD=0.03 g) and 4.64 g (SD=0.03 g), respectively.

**Figure 1:**
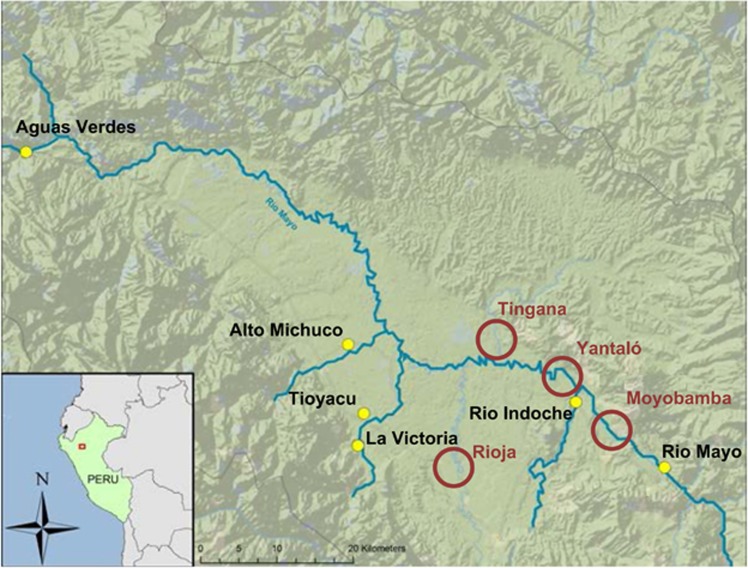
Alto Mayo region of Peru. Closed yellow circles (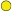) indicate the locations of stationary low-density polyethylene passive samplers that were deployed in 2013 (Supplementary Information). Silicone wristbands were distributed in four communities in 2014 (open red circles, 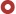). The Rio Mayo flows to the Southeast.

Volunteers were recruited in four communities of the Alto Mayo overlapping the areas targeted for water sampling (Figure 1). Institutional Review Board approval was obtained from three collaborating institutions: Medical College of Wisconsin, Oregon State University, and the Instituto Nacional de Salud of Peru. Informed consent was obtained from all participants. Moyobamba is the capital of the Department of San Martin with a population of >40,000. Rioja is an agriculturally oriented town and is home to approximately 20,000 people. Yantaló is a small farming village with fewer than 5000 people. Yantaló is also the home campus of the Yantaló Peru Foundation clinic, diagnostic center, and environmental health research base that formed the impetus for this study. Tingana is a small village with an ecological reserve and is an ecotourism destination, but many of its residents are also farmers. Each participant wore a wristband for 30–34 days in February and March, 2014. Demographic information collected from participants included community of residence, occupation, gender, and age. Wristbands were collected in their original PTFE bags, returned to Oregon State University, and coded with unique identifiers.

### Chemical Analysis

Each wristband was cleaned of particulate matter by rinsing with two rounds of 18 MΩ*cm water and once with isopropanol. To assess recovery during extraction of chemicals from wristbands, surrogate standards (TCMX, PCB-100, and PCB-209) were pipetted onto wristbands immediately before extraction. For extraction, each wristband was set in 100 ml of ethyl acetate in an amber glass jar for at least twelve hours at ambient temperature, the ethyl acetate (extract) was removed and the extraction was repeated with new ethyl acetate for two hours. For each wristband, the two extracts were combined and reduced to 1 ml.^8, 14^ Aliquots of the wristband extracts were spiked with the internal standard *p,p’-*dibromooctafluorobiphenyl and analyzed with gas chromatography. Extracts were diluted 1:10 to reduce analytical interferences of background lipids and siloxanes.

We screened for compounds using retention time locked full scan gas chromatography–mass spectrometry (GC–MS) paired with automated mass spectral deconvolution and identification system (AMDIS, National Institute of Standards and Technology, Gaithersburg, MD, USA) and deconvolution reporting software (DRS, Agilent Technologies, Santa Clara, CA, USA) as described elsewhere.^24^ The deconvolution package compares potential analytes to a list of PCBs, pesticides, PAHs, personal care products and pharmaceuticals (PCPPs), and more, which was compiled from commercially available mass spectral and retention time libraries (Agilent) and further expanded in-house to a total of 1397 compounds. The complete list of compounds is available in the Supplementary Information.

Wristband extracts were quantitatively analyzed for 63 pesticides and pesticide degradation products on a dual column gas chromatograph (Agilent 6890N) with dual micro-electron capture detection (GC-μECD, or “ECD”) as described in Donald et al.^14^ Of 63 pesticides, 46 are classified as insecticides, ten herbicides, and seven fungicides. Our method reflected interest in insecticides for their potential human health impacts and includes 30 organochlorines, six organophosphates, seven pyrethroids, three phenylpyrazoles, and one neonicotinoid. Three wristband extracts had backgrounds that obscured the internal standards in the ECD chromatograms so quantitative results are reported for the remaining 65 wristbands. Concentrations of pesticides in wristband extracts were corrected for dilution, surrogate recovery, and mass of the wristband. Final results are presented as ng/g wristband. Detection limits (Supplementary Information) were determined as described in Donald et al.^14^ and adjusted for mass of wristband and dilution. Method detection limits when using large wristbands were 2.88, 2.04, and 2.69 ng/g wristband for chlorpyrifos, cypermethrin, and DDx, respectively.

### Quality Assurance/Quality Control

Sample handling, analysis, and quantitation were performed as defined by laboratory data quality objectives and standard operating procedures. As trip blanks, two constantly sealed wristbands accompanied researchers during deployments and one field blank was exposed to the air at Rioja and Tingana each. Laboratory procedural blanks in the form of non-deployed wristbands were concurrently cleaned and extracted with deployed samples. These quality control samples were analyzed concurrently with deployed samples. Surrogate standard compounds accounted for any loss during extraction and analysis of PSDs. Instrument blanks and continuing calibration checks were analyzed regularly during chromatography. During ECD analysis, the formation of *p,p′-*dichlorodiphenyldichloroethane (DDD) from *p,p′-*dichlorodiphenyltrichloroethane (DDT) was observed in some calibration check samples so DDT and its metabolites *p,p′-*dichlorodiphenyldichloroethylene (DDE) and DDD are reported as the sum of the three related compounds (DDx).^26^ Samples were randomized for chemical analysis, and data was collected blind to the sample identity.

Average surrogate recoveries (relative standard deviation) were 76% (36%) for TCMX, 75% (27%) for PCB-100, and 99% (17%) for PCB-209. All laboratory and trip blanks were free of pesticides during GC-ECD analysis. Two pesticides were measured in the field blanks (Tingana: 12 ng DDT/g wristband; Rioja: 17 ng chlorpyrifos/g wristband). Given the absence of these compounds in the rest of the QC samples, and that they are two of the most frequent and abundant compounds at the respective deployment locations, the presence of chlorpyrifos and DDT likely reflect the field blanks’ brief exposure to external contamination in the environment. Similarly, DEET was detected in one field blank with the GC–MS screen but was not omitted. Some phthalates were detected in field and laboratory QC, highlighting pervasive background contamination of these compounds in the field and lab. However, the relative abundances in deployed wristbands were orders of magnitude higher than in QC samples, so they were included in analyses.

### Data Analysis

Statistical analysis was performed with the software package JMP Pro 12 (SAS, Cary, NC, USA). The 106 chemicals detected in wristbands with the GC–MS screen were grouped into eight chemical classes before analysis. Ideally, compound classification would be based on known chemical application in the participating region, but it is impossible to collect that information for hundreds of chemicals *a priori*. We aimed to be as robust as possible by assigning chemical class before performing analyses and by adjusting the significance level for multiple comparisons. The proportion of those chemicals detected in wristbands from each demographic group was compared for each class (Supplementary Equation 1). Chi-square likelihood ratio test was used to compare between the four communities and Fisher’s Exact Test was used for comparisons of gender and occupation. The *P*-values were compared with a Bonferroni adjusted significance level for eight chemical classes, *α*=0.05/8=0.0063. Because there were very few fungicides detected in some communities, the Chi-square test was suspect so Fisher’s Exact Test was also performed for the 4 × 2 contingency table of fungicides and community yielding the same results (data not shown).

Because chlorpyrifos, cypermethrin, and DDx were quantified in more than 50% of the wristbands, we explored the effects of community, occupation, gender, and age on these pesticides. With either zero or the detection limit substituted for non-detects, the concentrations of chlorpyrifos and DDx were log-normally distributed and cypermethrin had a bimodal distribution. Observations below the detection limit made up approximately 9%, 29%, and 3% of the data for chlorpyrifos, cypermethrin, and DDx. For analysis, non-detects were set to zero ng/g and one-sided nonparametric statistical tests were used. Differences in the median concentration of each pesticide between communities were assessed using analysis of variance with Wilcoxon rank sum for multiple comparisons. Wilcoxon rank sums test was used for occupation and gender. Variance was determined to be equal for all comparison groups by Levene’s Test for chlorpyrifos and DDx, and by the Brown-Forsythe test for cypermethrin. The effect of age for each pesticide was evaluated with a simple linear regression with a robust model fit using the Huber M-estimation method. Test statistics were assessed at a Bonferroni adjusted significance level for the three analytes, *α*=0.05/3=0.0167. These comparisons were also performed with parametric tests after substituting zero or the limit of detection for non-detects, or omitting the non-detects altogether. Some differences in the significance were found but the overall conclusions did not change.

To account for interactions between demographic variables, we constructed multiple linear regressions of chlorpyrifos, cypermethrin, and DDx. Concentrations were left-centered so the natural log of the wristband concentration was used in the standard least squares models. Community, occupation, gender, and age were used to build models using Moyobamba, non-farm workers (other), and females as reference levels. Model terms were added and subtracted to minimize Akaike’s information criterion. During initial model assessment, non-detects were identified as influential outliers, even when substituting the detection limits. These cases were omitted, and we restricted our analysis and inference to cases where concentrations in wristbands were above detection limits. Six, 19, and two cases were removed for regression of chlorpyrifos, cypermethrin, and DDx, respectively. Major conclusions were the same with and without non-detects (Supplementary Table 5).

To estimate relative time since application of DDT, the ratio of *p,p′-*DDT/(*p,p′-*DDE + *p,p′-*DDD) was calculated as first suggested for wristbands by Donald et al.^14^ and follows similar diagnostics in environmental media^27^ and human biomonitoring.^28, 29^ The DDT ratios of different communities were compared with Wilcoxon sum rank tests.

## Results

### Environmental Passive Sampling

Chemicals detected in the surface water of the Alto Mayo are presented in the Supplementary Information including Supplementary Table 1 and Supplementary Figure 1.

### Compliance and Participant Demographics

Sixty-nine wristbands were returned to researchers. One wristband was returned without a demographic survey, so data analysis was performed on 68 complete sets of paired wristbands and surveys. Participant demographics are provided in Table 1. Participants most frequently reported “farm worker” in their occupation description (25/68). Other occupations included housewife (16/68), student (12/68), and public employee (4/68).

### 1397 Compounds by GC–MS Screen

**Figure 2:**
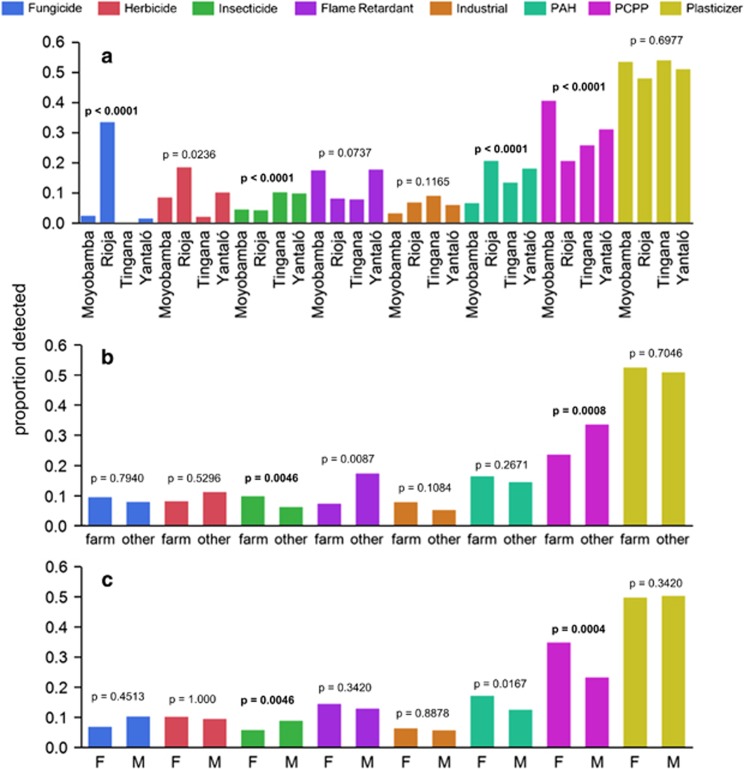
Proportion of positive detections for 8 chemical classes detected in silicone wristbands worn by residents of the Alto Mayo. Differences between communities (**a**) were evaluated with chi-square likelihood ratio test: *n*=15, 15, 13, and 25 for Moyobamba, Rioja, Tingana, and Yantaló, respectively. Gender (**b**) and occupation (**c**) were compared with Fisher’s Exact Test. *P*-values in bold are less than the significance level of 0.0063, adjusted for multiple comparisons. farm: farm worker (*n*=25), other (*n*=43), M: male (*n*=33), F: female (*n*=35). The proportions are out of three fungicides, four herbicides, 26 insecticides, five flame retardants, 13 industrial compounds, 29 PAHs, 15 PCPPs, and 11 plasticizers.

A total of 106 unique compounds were detected among 68 wristbands. The most commonly observed compounds, in almost every sample, were four phthalate acid esters and two musks galaxolide and tonalide. All compounds were grouped into one of eight classes: three fungicides, four herbicides, 26 insecticides, five flame retardants, 13 industrial compounds, 29 PAHs, 15 PCPPs, and 11 plasticizers. The full list of detected compounds is provided in Supplementary Table 2. Figure 2 shows the distribution of each chemical class between communities, gender, and occupation.

**Figure 3:**
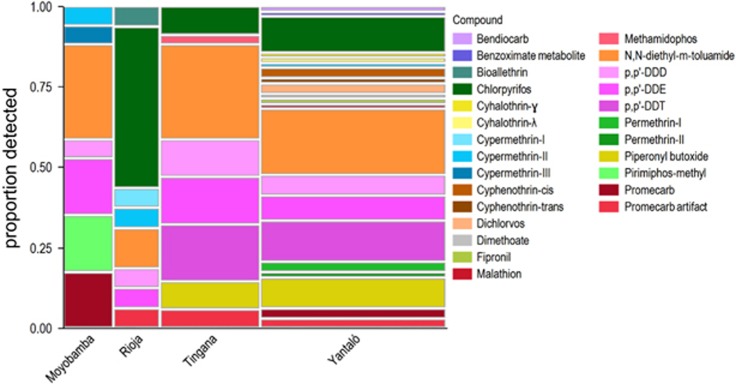
Relative contribution of individual insecticides to the total number of detections in silicone wristbands worn in each of four communities in the Alto Mayo. The width of the columns is proportional to the number of detections in wristbands from that community; Moyobamba: 17 detections in 15 wristbands, Rioja: 16 detections in 15 wristbands, Tingana: 34 detections in 13 wristbands, and Yantaló: 63 detections in 25 wristbands. The height of each colored box represents the proportion that an individual compound contributes to the total detections in that community. Isomers of cypermethrin and permethrin are not specified in the DRS library so are listed in retention order with roman numerals.

Figure 3 displays the profile of insecticides in each community. Additionally, the fungicides trifloxystrobin and tebuconazole were detected in seven of the 15 wristbands from Rioja, with both usually detected in the same wristbands. While herbicides were not significant as a class, nine of 15 Rioja wristbands contained butachlor comprising 72% of the herbicide detections across the study. PAHs were detected in greater proportions in wristbands from Rioja, Tingana, and Yantaló than Moyobamba. In contrast, samples from Moyobamba had the greatest proportion of PCPPs, seemingly due to more detections of musk ketone, benzophenone, and benzyl benzoate.

In confirmation of the above results, a hierarchical cluster analysis identified six clusters of samples (Supplementary Figure 2) that largely resembled differences identified in Figure 2. Together these results demonstrate patterns of potential chemical exposure between groups.

### Pesticides by ECD

Among compounds common to both the GC–MS screen and ECD methods, we identified 61 of the same detections. We identified 532 more pesticide peaks in the ECD chromatograms and only three peaks were identified solely by the GC–MS screen. In all, 16 pesticides (15 insecticides and one herbicide) were identified among 65 wristband samples. Three compounds were detected in more than 50% of wristbands: chlorpyrifos (59/65 wristbands, 17–9000 ng/g), cypermethrin (46/65 wristbands, 77–7000 ng/g), and DDx (63/65 wristbands, 8.8–5400 ng/g). Other compounds measured in wristbands were (in decreasing frequency) fipronil sulfide, dieldrin, dacthal, deltamethrin, permethrin, dimethoate, prophos, o,p’-dicofol, *β*-hexachlorohexane, *α*-chlordane, endosulfan-I, fipronil sulfone and *λ*-cyhalothrin. The quantitative method did not target DEET which was commonly detected in the GC–MS screen.

**Figure 4:**
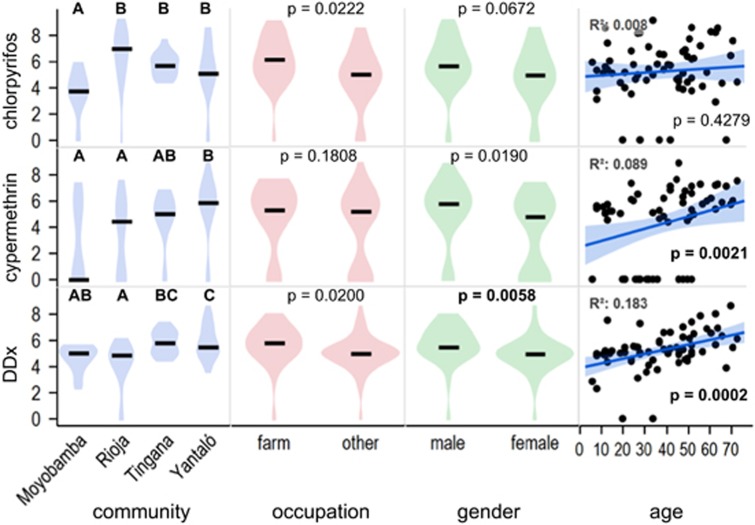
Differences in *ln*(concentration +1) of the most common and abundant pesticides measured in silicone wristbands between major demographic groups in the Alto Mayo. Horizontal bars indicate the median and shaded regions represent the distribution of the data. Community was compared using ANOVA with Wilcoxon rank sum tests; letters connect groups that are not statistically different (for statistical details, see Supplementary Table 4). Occupation and gender were compared with Wilcoxon rank sums test, and the slope of age was assessed with a robust line of best fit. *P*-values are bold if lower than the adjusted significance level of 0.0167.

Differences between demographics for the three most common pesticides are presented in Figure 4 and results of optimized multiple linear regressions in Table 2. Accounting for everything else, age had the most significant effect on cypermethrin and DDx concentrations but was not associated with chlorpyrifos. Occupation had no effect on the concentrations of these compounds, although for chlorpyrifos, there was a significant interaction between occupation and community. Gender was not a significant parameter for any of the three models, but it was included in the optimized model for DDx, probably due to being near the 0.05 significance level. After optimization, model fits were better than age by itself but did not surpass an *r*^2^ value of 0.40.

DDT ratios ranged from 0 to 9.5 (Supplementary Figure 3). Thirty-one wristbands (48%) had DDT ratios greater than one, reflecting relatively recent DDT exposure. The median DDT ratio in wristbands from Tingana was significantly greater than each of the other communities (Wilcoxon sum rank test, alpha=0.05). While the median DDT ratio in Yantaló wristbands was less than one, several participants had elevated ratios suggesting mixed sources of DDT in that community.

## Discussion

The return rate of wristbands (92%) was quite good considering that one month is the longest wristbands have been worn for a study. Similar compliance success with silicone wristbands has been documented in studies with shorter deployment periods.^14, 15^ Participants were well distributed in gender and age but those from Tingana and Moyobamba were either all “farm worker” or “other,” respectively. Moyobamba is the capital of the Department of San Martin and the largest community in this study. Residents of Moyobamba may have more diverse occupations than small farming villages like Tingana. Therefore, occupations of participants in this study may reflect real differences between communities.

### Distribution of Diverse Chemicals among Wristbands

When grouped by chemical class, patterns of exposure appear among the wristbands (Figure 2). The strongest differences were observed between communities, suggesting regional patterns of exposure. Relatively low numbers of PAHs and a high proportion of PCPPs distinguished wristbands worn in Moyobamba from the other communities. As the largest city in this study, the pattern of chemical classes detected in Moyobamba wristbands may represent an urban signature of the Alto Mayo. The other three communities had more PAHs and pesticides than Moyobamba which may generally represent rural exposures.

The most dramatic difference between communities was the detection of the fungicides trifloxystrobin and tebuconazole in wristbands from Rioja. Trifloxystrobin and tebuconazole are commonly used together in several pesticide formulations registered for use on rice in Peru.^30^ Although not significant, Rioja also had markedly more hits of herbicides than other regions, the majority of which were butachlor. Participants with these compounds were of mixed gender and occupation so more information is necessary to understand which community members in Rioja were handling these chemicals.

More insecticides were detected in wristbands from Tingana and Yantaló than the other communities. The profile of specific insecticides detected in wristbands from each community may reflect different use patterns (Figure 3). DEET, DDE, and DDD were detected in every community. DDT and piperonyl butoxide were only detected in wristbands from Tingana and Yantaló. Chlorpyrifos was detected at the highest rate in Rioja wristbands (50% of insecticides in Rioja compared with 8.8% and 11% in Tingana and Yantaló, respectively) but was never detected in Moyobamba. Pirimiphos-methyl was uniquely identified in Moyobamba. We speculate that Moyobamba may use some pesticides, such as pirimiphos-methyl, for residential pest control, while the agricultural communities of Rioja, Tingana, and Yantaló may use chlorpyrifos occupationally. Indeed, farm workers’ wristbands had more insecticides than other occupations (Figure 2).

Wristbands worn by non-farm workers contained more PCPPs than wristbands worn by farmers. Nineteen of the 25 farm workers were men, so insecticides and PCPPs were also significantly different between genders. Previous studies showed greater concentrations of benzophenone in urine of women than men,^31, 32^ which supports the generalization that PCPPs such as perfumes and lotions are more commonly used by women. Regardless of gender, more PCPPs were detected in wristbands from Moyobamba than other communities (Supplementary Figure 4). Although not significant, we also observed more flame retardants in wristbands worn by non-farm workers. Therefore PCPPs, and potentially flame retardants, may also be chemical signatures of urban living in the Alto Mayo.

We observed weak evidence (*P*=0.0167), although not significant, that women had more PAHs in their wristbands than men (Figure 2). By examining each region individually, we observed more PAHs in wristbands of women than men from Rioja and Yantaló but not in Moyobamba or Tingana (Supplementary Figure 5). PAHs can come from many sources but one gender-specific hypothesis is that women are more likely to work with cooking fires resulting in disproportionate exposure to PAHs.^33, 34^ Future work could determine source profiles (e.g., pyrogenic or petrogenic) of PAHs in air and wristbands to explore differences in sources of exposure in the Alto Mayo.

Plasticizers were the most commonly detected compounds in all groups. Small amounts of phthalates may come from laboratory contamination or the wristband itself, but chromatographic responses of phthalates were 100 to 10,000 times greater in deployed samples than trip, field, or laboratory blanks. Phthalates have been measured in skin wipes,^35^ as metabolites in urine,^36^ and nails,^37^ and identified as some of the most pervasive environmental contaminants.^38^ Perhaps because of pervasive phthalate exposure, we did not observe any trends in the detection of these compounds. Quantitative assessment of phthalates may resolve differences between groups.

Hierarchical clustering identifies closely related samples without forcing associations between samples or analytes. Therefore, several of the major chemical groups identified in Figure 2 also clustered naturally (Supplementary Figure 2). For example, wristbands from Rioja that contained the fungicides trifloxystrobin and tebuconazole were clearly distinguished. Wristbands worn in Moyobamba only grouped in two of the six clusters. Those clusters were generally characterized by either PCPPs or the absence of defining chemicals. Two clusters of wristbands that seemed to have the most PAHs were comprised of mostly female non-farm workers from Tingana, Yantaló, and Rioja. A group of mostly male farm workers from Tingana and Yantaló that clustered together were more likely to have DDT, DDE, DDD, and piperonyl butoxide, a finding corroborated in Figure 3. These results further demonstrate chemical signatures associated with lifestyle.

### Distribution of Individual Pesticides

Of the compounds studied here, pesticides may pose the greatest health risks to residents in the Alto Mayo because of potentially high exposures and specific toxic modes of action. For example, chlorpyrifos and other organophosphate insecticides can be acutely toxic through acetylcholinesterase inhibition,^39^ and DDT, dieldrin, hexachlorohexane, chlordane, and endosulfan are listed as banned or restricted persistent organic pollutants by the Stockholm Convention, which Peru has ratified.^40^ Additionally, because pesticides are intentionally applied, they might be the most manageable chemical exposure through education and improving access to personal protection equipment.^22, 41, 42^ Therefore, we further evaluated the distribution of pesticides among the wristbands with the most frequently detected pesticides, chlorpyrifos, cypermethrin, and DDx.

Some trends observed in the screening results were also apparent for the masses of chlorpyrifos, cypermethrin, and DDx in wristbands. For example, Moyobamba tended to have the lowest concentrations of those pesticides, even after accounting for other variables (Table 2). Occupational differences were not significant for the top pesticides (Figure 4), although chlorpyrifos and DDx would be significantly greater in farmer’s wristbands at a more lenient significance level. We observed no differences between genders after controlling for occupation, age, and community (Table 2). Sampling with silicone wristbands targets contaminants in the external environment so, while they may pick up some compounds from skin, is probably not sensitive to biological processes such as maternal transfer.^43^

Overall, broad demographic factors explained a surprising amount of differences in chemical exposure, but while we were able to explain up to 40% of the variability (*r*^2^≤0.40), most still remained unexplained. Naturally, a person’s exposure is not only determined by the size of her community or her occupation but also many other factors that we did not assess in this study. Participants who did not self-describe as a farm worker may have also helped to mix or apply pesticides, and exposure could be distributed through pesticide handling and storage,^22, 23^ and medicinal uses.^44^ Anecdotes from Tarapoto, near the Alto Mayo, reported that pesticides are often stored in the home,^18^ as has been documented for rural farmers in Africa,^22, 23^ and that other family members will help mix and apply pesticides.^19^ It is also possible that pesticides applied domestically would confound differences between occupations.

Consistent with screening results that show more insecticides with increasing age (Supplementary Figure 6), cypermethrin and DDx were significantly associated with age (Figure 4) although correlation coefficients were low. Correlation of wristband concentrations with age may reflect legacy contamination. Lasting exposure to lipophilic chemicals could accumulate in skin and be a source to the wristband as O’Connell et al.^8^ postulated. However, for chemicals that are readily metabolized, such as chlorpyrifos, we would not expect to see bioaccumulation^45^ or, therefore, an association with age. Pyrethroids and DDT can be used in topical medicines, for example to treat scabies,^44^ so there is potential for especially extensive dermal contact which could accumulate over time. DDT has a long history of use in Peru for malaria control but ceased *circa* 1990.^21^ Lange^18^ detected DDE, the main metabolite of DDT, in the blood of farm workers of Tarapoto, a city near the Alto Mayo. The DDE plasma concentrations were also correlated with the age of participants. Because DDT itself was not detected in blood, Lange concluded that DDT is no longer used in the region but persists in the tissue of farm workers.^18^

The diagnostic ratio of DDTs in wristbands suggested there were recent applications of DDT in the Alto Mayo. Participants from Tingana and some participants from Yantaló had DDT ratios much greater than one (Supplementary Figure 3), indicating relatively recent application of DDT. Tingana and Yantaló are neighboring villages, so some community members may share exposures that the Rioja and Moyobamba do not. This forensic technique was first applied to silicone wristbands by Donald et al.^14^ who noted minor differences in sampling rate between DDT and its metabolites. Degradation of DDT during GC analysis could also influence the DDT ratio,^26^ an artifact that we minimized with randomized sample analysis. Different sampling rates and decomposition at the instrument would both decrease the apparent DDT ratio so, if anything, bias the results toward a historical signature and our conclusions would not change: wristbands from Tingana had the greatest DDT ratios, suggestive of recent application.

### Regional Context

To get an overview of what pesticides and other compounds might be present in the environment in the Alto Mayo, we conducted an initial survey of bioavailable contaminants in the surface water of the Alto Mayo in August 2013. Details on methods and results are presented in the Supplementary Information. Many of the compounds detected in wristbands were also identified in surface water. Chlorpyrifos, DDTs, cypermethrin, *λ* -cyhalothrin, butachlor, tonalide, cashmeran, biphenyl, sulfur, and PAHs were measured at sampling locations downstream of significant agriculture, *i.e.* every location except Aguas Verdes. The fungicide cyclafuramid was the only compound detected in surface water but not in wristbands. Chlorpyrifos was especially pervasive in the Alto Mayo as it was the most concentrated pesticide in every water sample downstream of agriculture and in 91% of wristbands. This likely reflects widespread application of chlorpyrifos for agricultural or domestic pest control.

Several of the pesticides detected in this study were reportedly used in the nearby city of Tarapoto including tebuconazole, cypermethrin, methamidophos, permethrin, and butachlor.^18^ Chlorpyrifos was not among the chemicals reportedly used in Tarapoto and it was not a target analyte in analyses of surface water and blood samples there.^18, 19^ Most pesticides detected in the current study, including chlorpyrifos but not DDT, are registered for agricultural use in Peru.^30^ However, some pesticides including DDT may be used as medicine or to control the malaria vector and it is possible that some pesticides are introduced to the Alto Mayo illegally. Donald et al. found that approximately half of the pesticides detected in silicone wristbands worn by West African farmers were reportedly applied^14^ so we expect to observe chemicals not explicitly used. Additionally, Donald et al. found many of the same compounds as reported in the current study (including cypermethrin as one of the most commonly detected). However, chlorpyrifos and DDT were detected in less than 50% of wristbands. Instead, deltamethrin, and *λ*-cyhalothrin were more prevalent compounds in West Africa.^14^

### Wristband Applications and Limitations

As passive sampling devices, silicone wristbands sample organic compounds in a partition-based method, similar to partitioning into lipid tissue. Because each chemical has its own affinity for the silicone polymer, we intentionally refrained from comparing the presence or concentrations of different chemicals. However, for compounds with similar chemical-specific partitioning to an organic phase we may be able to perform simple comparisons. The most commonly detected compounds, chlorpyrifos, cypermethrin, and DDT, have logK_ow_s of 4.96, 6.60, and 6.91 respectively, and logK_oa_s of 8.88, 10.8, and 10.38, respectively. With a logK_ow_ of 6.8 and logK_oa_ of 11.3, *λ*-cyhalothrin has a similar affinity for silicone wristbands but was only detected in a few cases. Therefore we infer that participants in this study were more commonly exposed to chlorpyrifos, cypermethrin, and DDT than they were to *λ*-cyhalothrin. Methamidophos, in contrast, has a logK_ow_ of −0.80 and a logK_oa_ of 6.65. Methamidophos was reportedly used in nearby Tarapoto^18^ and an empty bottle was observed in the field during this study but was only detected in one of 68 wristbands in the Alto Mayo. Methamidophos is likely more pervasive than the wristbands were able to detect.

Silicone wristbands, as deployed in this study, are multi-media samplers. Therefore we cannot infer to specific routes of exposure and provided both air and water partitioning coefficients as examples. We assume that internal exposure is underestimated because wristbands do not account for ingestion, an important route of exposure to chemicals such as organophosphate pesticides.^39^ Finally, the current work was limited in the number of participants and relied on non-random recruitment so we cannot infer to the population level.

## Conclusions

Using silicone wristbands, we can identify chemical mixtures in the personal environment that reflect lifestyle of participants, and we are able to infer about potential sources of exposure. These multi-class profiles are important identifiers of typical mixtures that people of similar lifestyle would experience. Silicone wristbands are an excellent complement to health research, especially in remote regions where practical constraints might hinder use of other techniques. Future work can use wristbands to associate elevated wristband exposure with adverse health effects such as between insecticides and acute pesticide poisoning or neurobehavioral development.^3^ More development is needed to relate wristband results to environmental concentrations and internal exposure.

## Figures and Tables

**Table 1:** Major demographics of participants who wore silicone wristbands.

*Location*	n	*Gender*		*Age*			*Occupation*	
		*Female (%)*	*Male (%)*	*Min.*	*Median*	*Max.*	*Farm worker (%)*	*Other (%)*
Moyobamba	15	66.7	33.3	6	36	63	0.0	100.0
Rioja	15	53.3	46.7	12	28	73	33.3	66.7
Tingana	13	30.8	69.2	19	50	59	100.0	0.0
Yantaló	25	52.0	48.0	8	45	70	28.0	72.0
Total	68	51.5	48.5	6	39	73	36.8	63.2

**Table 2:** Effect estimates from multiple linear regressions modeling the concentration of three pesticides in wristbands (ng/g wristband).

	*Parameter*^a^	*Estimate*	*SE*	P*-value*^b^	r*^2^*
*ln*(chlorpyrifos)	Intercept	5.45	0.21	**<0.0001**	0.39
	*Community*				
	Yantaló-Moyobamba	0.78	0.33	**0.0221**	
	Tingana-Moyobamba	0.52	0.56	0.3645	
	Rioja-Moyobamba	1.30	0.31	**<0.0001**	
	Occupation (farm worker)	−0.26	0.36	0.4835	
	Occupation*Community	1.10	0.47	**0.0239**	
					
*ln*(cypermethrin)	Intercept	5.08	0.30	**<0.0001**	0.30
	*Community*				
	Yantaló-Moyobamba	0.41	0.20	**0.0497**	
	Tingana-Moyobamba	−0.51	0.24	**0.0415**	
	Rioja-Moyobamba	−0.23	0.27	0.3854	
	Age	0.02	0.01	**0.0056**	
					
*ln*(DDx)	Intercept	4.37	0.30	**<0.0001**	0.40
	*Community*				
	Yantaló-Moyobamba	0.54	0.19	**0.0071**	
	Tingana-Moyobamba	0.29	0.25	0.2341	
	Rioja-Moyobamba	−0.39	0.24	0.1148	
	Gender (male)	0.24	0.13	0.0746	
	Age	0.02	0.01	**0.0016**	

a The parameters community, occupation, gender, and age were used in building these regressions. Reference level is female non-farm workers from Moyobamba.

b *P*=*P*-value at significance level of 0.05. *P*-value is bold below significance level of 0.05.
