# Supplementary material for: Multi-class chemical exposure in rural Peru using silicone wristbands
Source: J Expo Sci Environ Epidemiol. 2017 Jul 26;27(6):560–8. doi: 10.1038/jes.2017.12 (PMC5658680; doi:10.1038/jes.2017.12)
Supplement: Supplementary Information [file jes201712x1.docx]

Multi-class chemical exposure in rural Peru using silicone wristbands

Supplemental information

Alan J. Bergmann, Paula E. North, Luis Vasquez, Hernan Bello, Maria del Carmen Gastañaga Ruiz, Kim A. Anderson

Note: Table S2 is a separate document.

Table of Contents.

[Information about stationary environmental passive sampling in the Alto Mayo 2](#_Toc476227895)

[Table S1. Chemicals detected in water of Alto Mayo. 4](#_Toc476227896)

[Table S3. Limits of detection and limits of quantitation for analytes quantified using GC-ECD. 5](#_Toc476227897)

[Figure S1. Chlorpyrifos measured in surface water of the Alto Mayo 7](#_Toc476227898)

[Equation S1. 8](#_Toc476227899)

[Figure S2. Hierarchical clustering of GC-MS screen results. 9](#_Toc476227900)

[Figure S3. DDT and its metabolites in four communities of the Alto Mayo. 10](#_Toc476227901)

[Figure S4. PCPP detections by gender and community. 11](#_Toc476227902)

[Figure S5. PAH detections by gender and community. 12](#_Toc476227903)

[Figure S6. Effect of Age on the detection rate of eight chemical classes in wristbands worn by residents of the Alto Mayo. 13](#_Toc476227904)

[Table S4. Statistical results of comparing community for the *ln*(concentration + 1) of the top three pesticides as seen in Figure 4. 14](#_Toc476227905)

[Table S5. Alternative multiple linear regression results. 15](#_Toc476227906)

References cited 17

List of analytes in GC-MS screen 18

Information about stationary environmental passive sampling in the Alto Mayo

To get an overview of what pesticides and other compounds might be present in the environment in the Alto Mayo, we conducted an initial survey of bioavailable contaminants in the surface water of the Alto Mayo in August 2013.

The LDPE passive sampling devices were prepared as described in Anderson et al. (1) LDPE tubing (2.5 cm wide by 70 um thick, Brentwood Plastic, Inc. St Louis, MO) were cut to 110 cm (4.82 ± 0.08 g).(2) The LDPE were placed in amber glass jars and conditioned to remove impurities with three rounds of soaking in hexane with gentle agitation for 48 hours. Clean LDPE was dried under vacuum for 48 hours, placed in pre-cleaned air-tight metal cans and stored at -20°C until deployment preparation. To prepare the LDPE strips for deployment, a loop was heat-sealed at one end of the strip, performance reference compounds (PCB65-d5, PCB115-d3, PCB156-d3) were added to the inter-membrane space, air removed, and the open end sealed with a loop to a final length of approximately 100 cm. Prepared LDPE were sealed in PTFE bags and stored at -20°C until transport to the field. LDPE were deployed in water on steel frames, five of which were housed in a steel cage (Environmental Sampling Technologies; St. Joseph, MO).

LDPE strips were deployed at six locations in the Alto Mayo region of Peru (Figure 1) for 4-6 days in August 2013. Sites were selected for a wide spatial distribution, variety in water body types, and potential contaminant sources. Aguas Verdes was the farthest upstream location, targeted for being in a region of low human impact. The samples at Alto Michuco were collected from an irrigation channel that drains rice fields and flows next to residences. La Victoria samplers were in an aquaculture pond that houses farmed tilapia in a small village. Tioyacu is a river downstream of agriculture and a major regional cement factory. Rio Indoche is near the town of Yantaló, downstream of various agriculture fields. Another sample was collected from the Rio Mayo, downstream of the locations mentioned above and downstream of Moyobamba. Triplicate sets of PSDs were placed at Alto Michuco and Rio Indoche. An additional deployment was repeated at Alto Michuco for 42 days beginning immediately after the triplicate samplers were retrieved. Recovered LDPE were sealed in PTFE bags and returned to Oregon State University for analysis. The LDPE had little visible biofouling. To remove any periphyton or particulates, the LDPE were cleaned in the laboratory by light scrubbing and sequential dousing in 1 N HCl, 18 MΩ*cm water, and two iterations of isopropanol. Dry LDPE were stored in amber glass jars at -20°C until extraction. Surrogate standards (TCMX, PCB-100, and PCB-209) were pipetted onto the LDPE strips immediately before extraction with 2 rounds, 12 hours followed by 2 hours, of 200 mL n-hexane. Extracts were reduced to approximately 1 mL with TurboVap closed cell concentrator (Biotage, Uppsala, Sweden), quantitatively transferred to conical centrifuge tubes, and reduced again to 1 mL.

GC-MS screen

LDPE extracts were screened with GC-MS for 1,182 compounds following methods described in Anderson et al.(3)

GC-ECD

Pesticides in LDPE extracts were analyzed with GC-μECD as described previously (3). The columns were 17MS and XLB, at 30 m by 0.25 mm by 0.25 μm (Agilent). Inlets were operated at 250˚C, with 2 μL injection volume and helium carrier gas. The oven program started at 110˚C for 1 min, then 4˚C/min to 300˚C, held for 10 min. Detectors at 320˚C with makeup gas held at 60 mL/min. Limits of quantitation are provided by Anderson et al.(3)

LDPE vs. wristband analysis

Collection and analysis of LDPE water samples preceded the wristband components by several months. Method upgrades during that time resulted in some differences in operating parameters and number of target analytes between the sets of samples. Four compounds were therefore detected in wristbands that were not among target compounds for water deployed LDPE: fipronil sulfide, fipronil sulfone, deltamethrin, and galaxolide.

Surface water concentrations

Water concentrations of pesticides measured quantitatively with GC-μECD were calculated using *in situ* calibration with performance reference compounds and methods described previously (3).

Surface water results:

Chemicals detected in surface water

Pesticides, personal care products, and PAHs were detected in LDPE samplers deployed in surface water of the Alto Mayo (main text, Table 1). No compound except for benz[a]anthracene, a PAH, was above detection limits at Aguas Verdes. Chlorpyrifos was measured at all of the other five locations and ranged from 0.14 to 1.9 ng/L (Figure S1). Chlorpyrifos was at very similar concentrations in sequential measurements at Alto Michuco (Figure S1) suggesting that chlorpyrifos may be relatively constant over time although more sampling would be needed to confirm. Other compounds included the pesticides cyclafuramid and butachlor and the fragrances tonalide and cashmeran.

Table S1. Chemicals detected in water of Alto Mayo.

| location | compound | method^1^ | concentration  (ng/L) |
| --- | --- | --- | --- |
| Aguas Verdes | PAHs*^2^* | DRS |  |
|  |  |  |  |
| Alto Michuco | chlorpyrifos  tonalide  *p,p’*-DDT^3^  *p,p’*-DDE^3^  *p,p’*-DDD^3^  cypermethrin^3^  λ-cyhalothrin^3^  cyclafuramid^3^  biphenyl^3^  PAHs*^2^* | ECD + DRS  DRS  ECD  ECD + DRS  ECD  ECD + DRS  ECD  DRS  DRS  DRS | 0.87  0.0068  0.0056  0.0053  0.092  0.032 |
|  |  |  |  |
| Rio Indoche^4^ | chlorpyrifos  PAHs*^2^* | ECD  DRS | 0.33 |
|  |  |  |  |
| Tioyacu | chlorpyrifos  tonalide  sulfur  cashmeran  PAHs*^2^* | ECD  DRS  DRS  DRS  DRS | 0.14 |
|  |  |  |  |
| La Victoria | chlorpyrifos  PAHs*^2^* | ECD + DRS  DRS | 1.9 |
|  |  |  |  |
| Rio Mayo | chlorpyrifos  tonalide  butachlor  PAHs*^2^* | ECD  DRS  DRS  DRS | 0.20 |

^1^ Detection using gas chromatography with electron capture detection (ECD) or gas chromatography with mass spectrometry and deconvolution reporting software (DRS) or both.

^2^ Various PAHs were detected at every site.

^3^ This compound was only measured in the 42 day deployment at Alto Michuco.

^4^ Sample exposed to air during deployment so the calculated value does not reflect true water concentration.Table S3. Limits of detection and limits of quantitation for analytes quantified using GC-ECD.

| Analyte Name | LOD (ng/mL)^a^ | LOQ (ng/mL)^a^ |
| --- | --- | --- |
| a‐BHC | 1.23 | 3.69 |
| Acetamiprid | 50.0 | 150 |
| a‐Chlordane | 1.14 | 3.42 |
| Alachlor | 1.69 | 5.08 |
| Aldrin | 1.38 | 4.15 |
| b-BHC | 1.50 | 4.51 |
| Bifenthrin | 1.14 | 3.42 |
| Captafol | 79.1 | 237 |
| Captan | 6.13 | 18.4 |
| Chlorobenzilate | 1.86 | 5.59 |
| Chloroneb | 1.54 | 4.63 |
| Chloropropylate | 1.98 | 5.95 |
| Chlorothalonil | 1.37 | 4.10 |
| Chlorpyrifos | 1.64 | 4.91 |
| cis‐permethrin | 1.65 | 4.95 |
| Cyhalothrin | 1.59 | 5.74 |
| Cypermethrin | 1.16 | 4.20 |
| Dacthal | 1.18 | 4.26 |
| d‐BHC | 1.06 | 3.18 |
| Deltamethrin | 5.88 | 17.5 |
| Diallate | 2.10 | 6.29 |
| Diazinon | 2.55 | 7.65 |
| Dieldrin | 1.06 | 3.18 |
| Dimethoate | 1.34 | 4.03 |
| Endosulfan sulfate | 1.14 | 3.41 |
| Endosulfan-I | 1.18 | 3.55 |
| Endosulfan-II | 1.03 | 3.10 |
| Endrin aldehyde | 1.63 | 4.89 |
| Endrin* | 2.93 | 8.79 |
| Endrin ketone | 1.51 | 4.54 |
| Esfenvalerate | 1.50 | 5.44 |
| Etridiazole | 3.44 | 10.3 |
| Fipronil sulfone | 0.82 | 2.46 |
| Fipronil | 0.90 | 2.69 |
| Fipronil sulfide | 1.33 | 3.98 |
| g‐Chlordane | 1.21 | 3.64 |
| Heptachlor | 1.86 | 5.57 |
| Heptachlor Epoxide | 1.17 | 3.51 |
| Hexachlorobenzene | 1.33 | 4.00 |
| Imidan | 2.22 | 6.66 |
| Isodrin | 0.95 | 2.84 |
| Lindane | 1.28 | 3.84 |
| Malathion + Fenitrothion | 0.98 | 2.94 |
| Methoxychlor | 1.83 | 5.49 |
| Metolachlor | 1.59 | 4.76 |
| Mirex | 1.25 | 3.74 |
| o,p'‐Dicofol | 1.12 | 3.36 |
| Oxadiazon | 1.31 | 3.92 |
| p,p'‐DDD | 1.16 | 3.47 |
| p,p'‐DDE | 1.18 | 3.55 |
| p,p'‐DDT | 1.53 | 4.59 |
| p,p'‐Dicofol | 2.24 | 6.73 |
| Pendimethalin | 1.34 | 4.03 |
| Pentachloronitrobenzene | 1.10 | 3.31 |
| Perthane-I | 1.25 | 3.75 |
| Propachlor | 1.59 | 4.77 |
| Propanil | 1.24 | 3.71 |
| Prophos | 1.33 | 3.98 |
| Simazine | 22.6 | 67.8 |
| Trans‐nonachlor | 1.12 | 3.36 |
| Trans‐permethrin | 1.38 | 4.15 |
| Trifluralin | 1.79 | 5.37 |

^a^ Units are ng/ mL wristband extract. Method detection limits were obtained by multiplying these values by 10 for sample dilution and dividing by the mass of wristband (see main text and Donald et al. (4) for more details).

Figure S1. Chlorpyrifos measured in surface water of the Alto Mayo using LDPE deployed for 4 days. Error bars represent 1 standard deviation from the mean as measured for replicates at Alto Michuco and Rio Indoche and estimated for the others based on the average relative standard deviation for the replicated sites. The checkered bar represents LDPE deployed at Alto Michuco for 42 days. Sites are arranged by their approximate order of upstream to downstream in the watershed. *Water concentrations at Rio Indoche samplers are rough estimates because samplers were not submerged for the whole deployment period. BDL: Below detection limits.

Equation S1.

The proportion of chemicals detected in wristbands from each demographic group are shown in Figure 2 and Figure S6. The proportions were compared for each class according to the following equation:

$$Proportion detected= \frac{x_{CG}}{n_{C}\times n_{G}}$$

Where $x_{CG}$ is the number of compounds detected for each class (*C*) in each demographic group (*G*), $n_{C}$ is the number of total compounds of each class that were detected in any sample, and $n_{G}$ is the total number of participants in each demographic group.


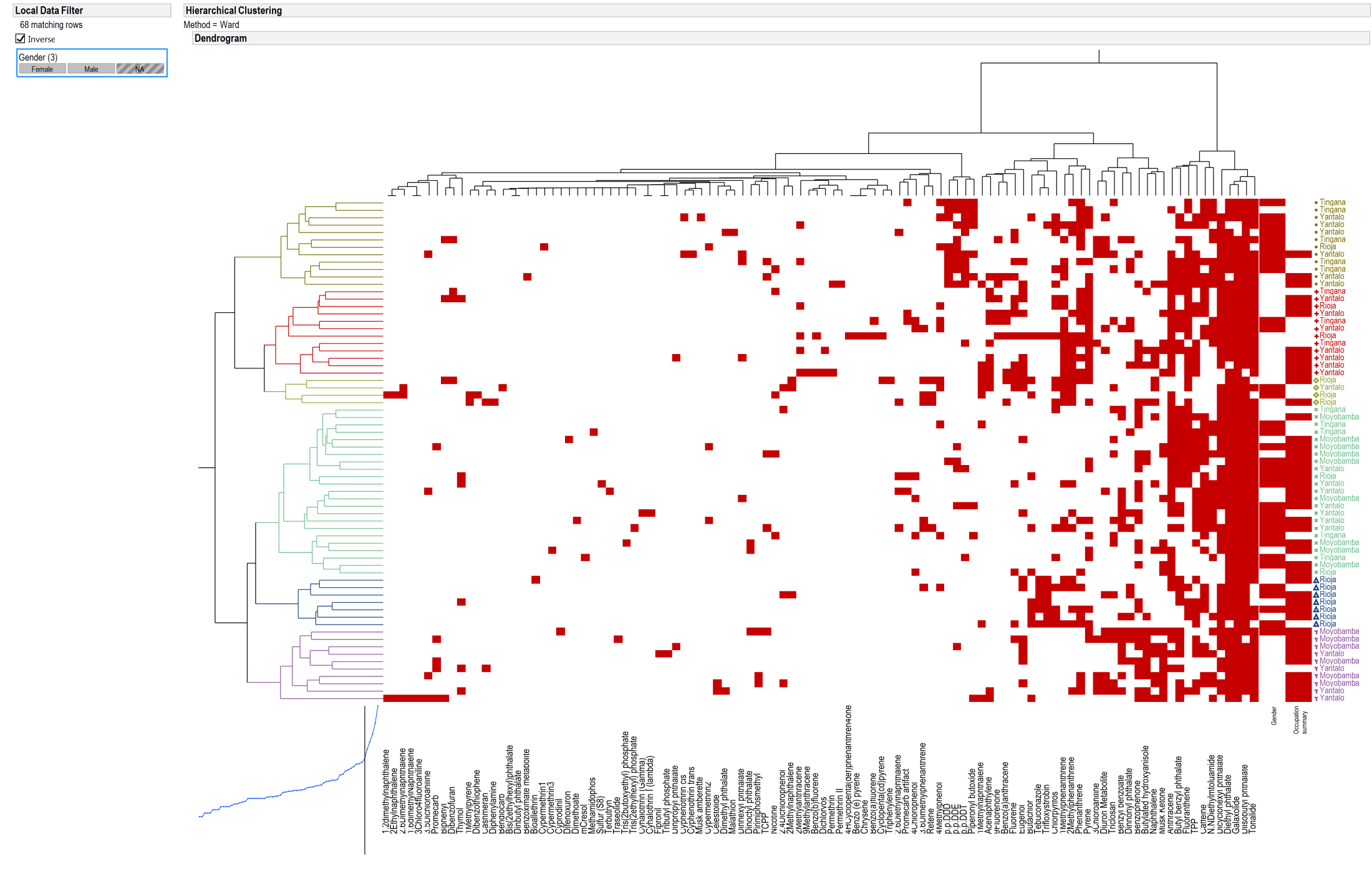


**fungicides/herbicides**

**PCPPs**

**DDTs**

**PAHs**

Figure S2. Hierarchical clustering of GC-MS screen results. Distance between observations was determined using Ward’s method (JMP Pro 12). Gender: red = male, white = female. Occupation: Red = Other, white = Farm worker. The clusters are colored in the community identifiers on the right axis and separated with solid black likes. The number of clusters was determined by analyzing the scree plot (bottom left of figure) for the natural break in the distance bridged by forming clusters. 2D clusters (black ovals) were labeled as the general class of compounds that make up the majority of the clusters. PCPP: personal care product and pharmaceutical. PAH: polycyclic aromatic hydrocarbon. DDTs: p,p’-dichlorodiphenyltrichloroethane and its metabolites.


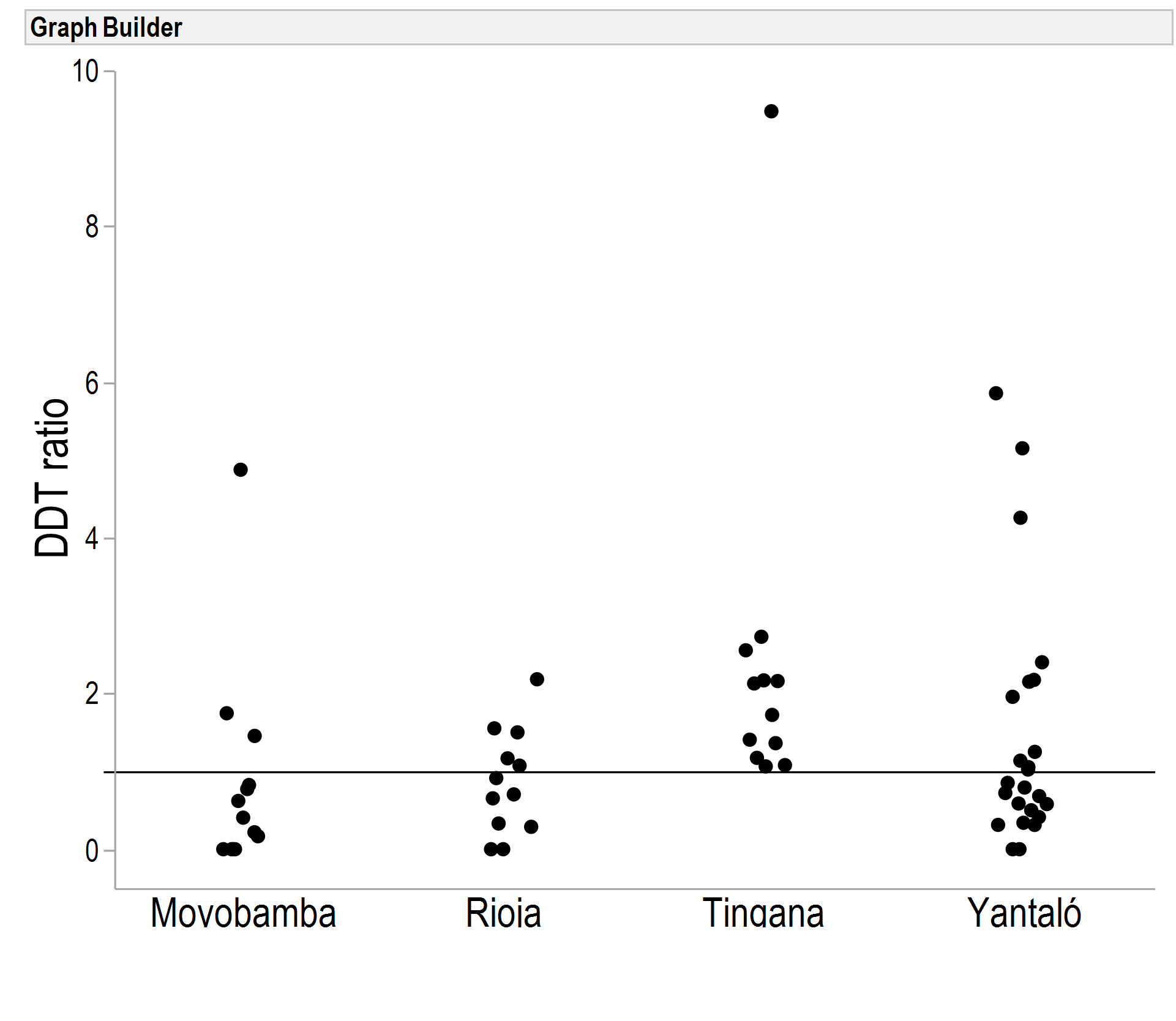


A


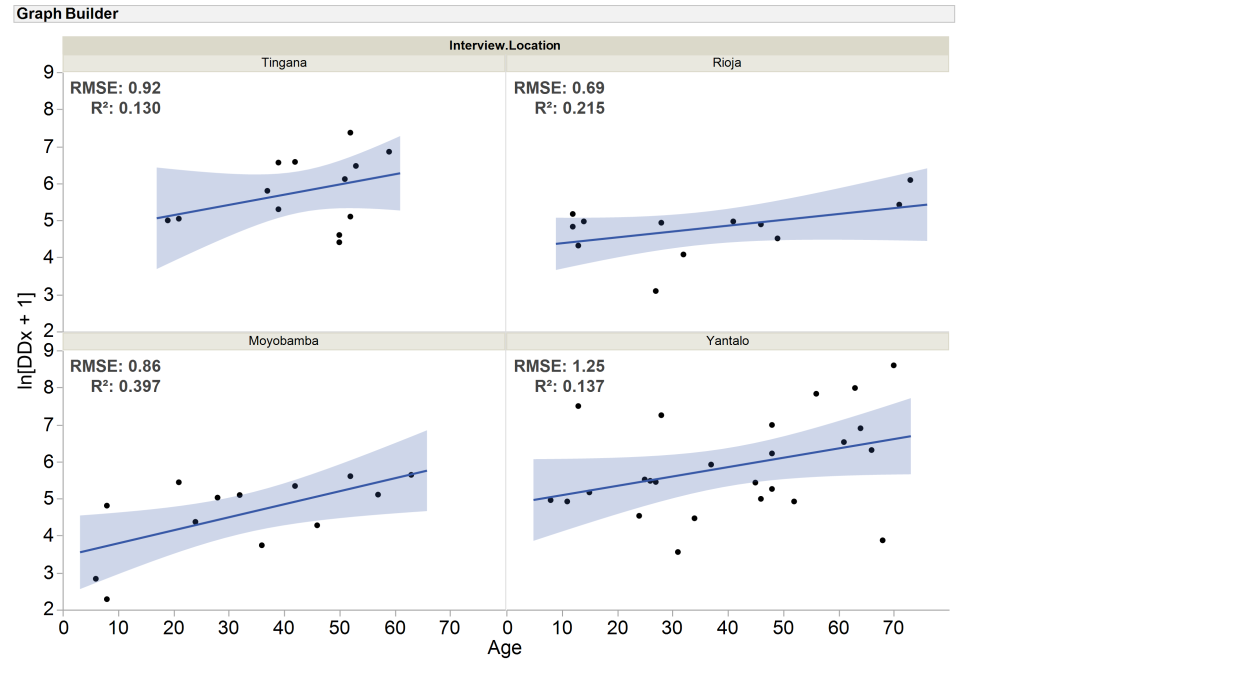


B

Figure S3. DDT and its metabolites in four communities of the Alto Mayo. (A) DDT ratio (DDT/[DDE + DDD]) greater than one indicates a relatively recent application of DDT. The solid horizontal line is a reference at DDT ratio = 1. (B) Correlations of DDx concentrations with age in each of the four communities. Shaded areas are the 95% confidence intervals. The concentration of DDx in wristbands from Tingana had the worst correlation with age of any community (B). In contrast, wristbands from the capital city, Moyobamba, contained the lowest DDT ratios and the greatest correlation with age. Rioja and Yantaló followed the same trend that high DDT ratios were associated with low correlations of wristband DDx concentrations with age. Together, these analyses support that an association with age indicates legacy contamination.


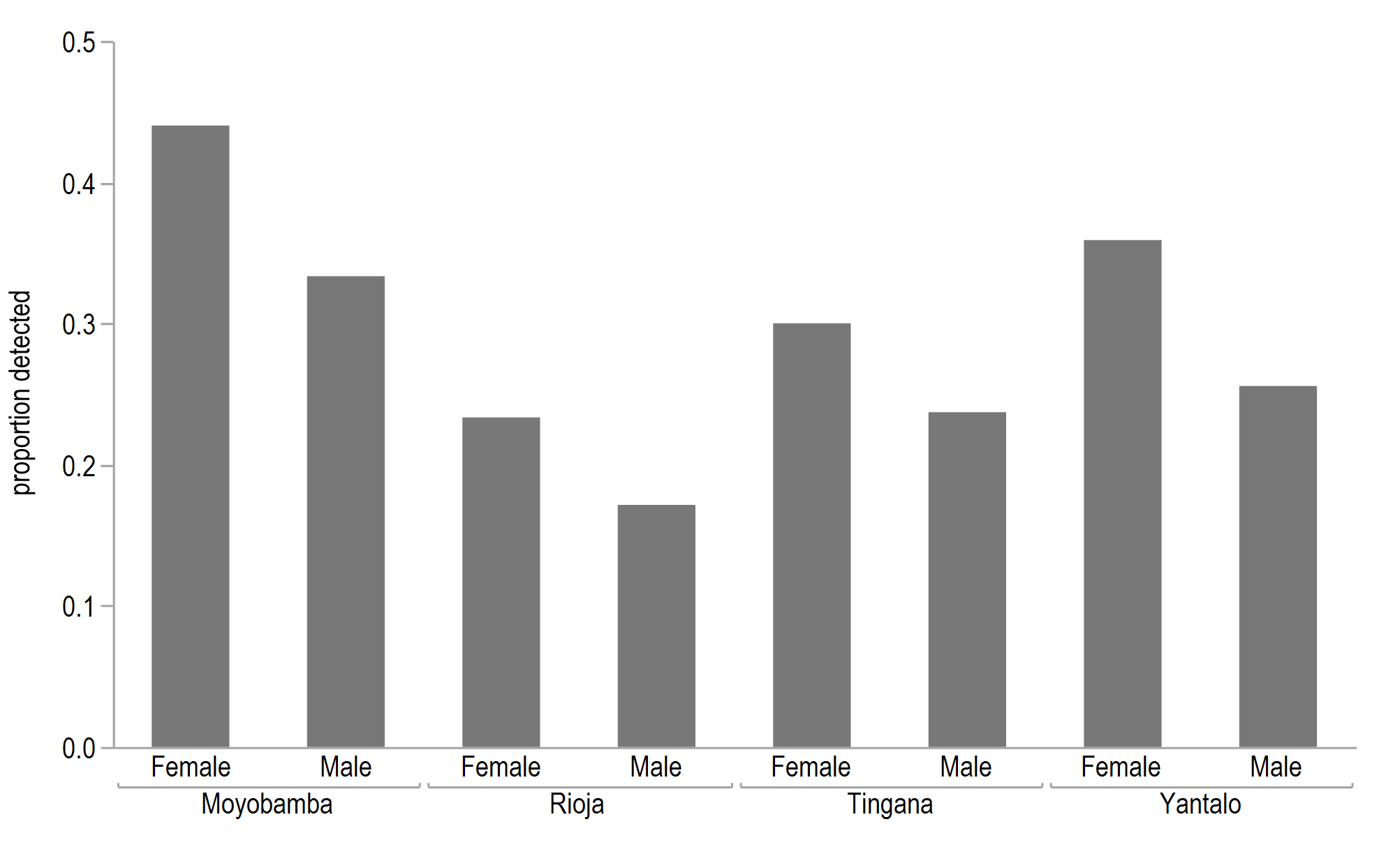


Figure S4. PCPP detections by gender and community. More PCPPs were detected in females’ wristbands than males’ in every community.


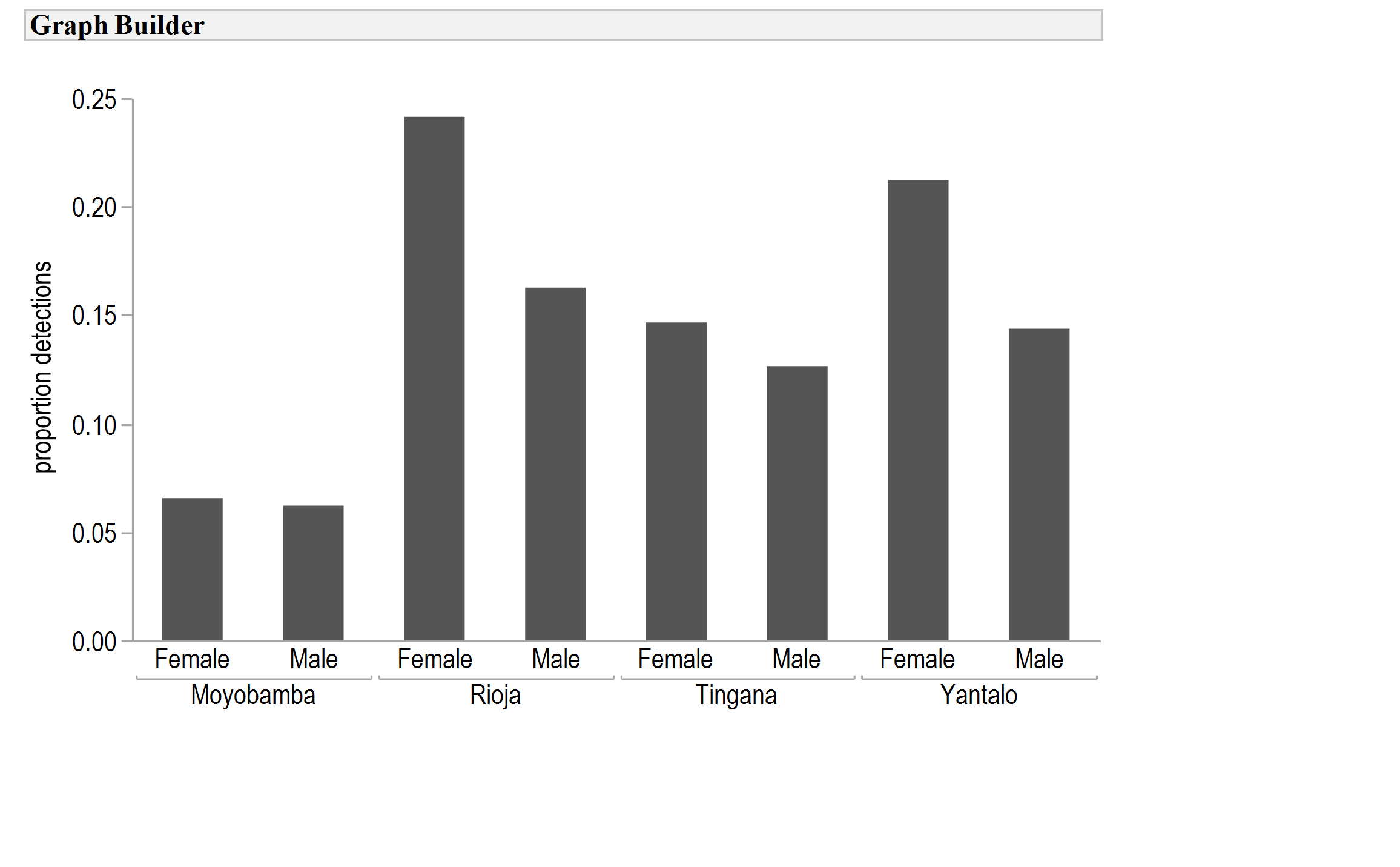


Figure S5. PAH detections by gender and community. More PAHs were detected in females’ wristbands than males, especially in Rioja and Yantaló.


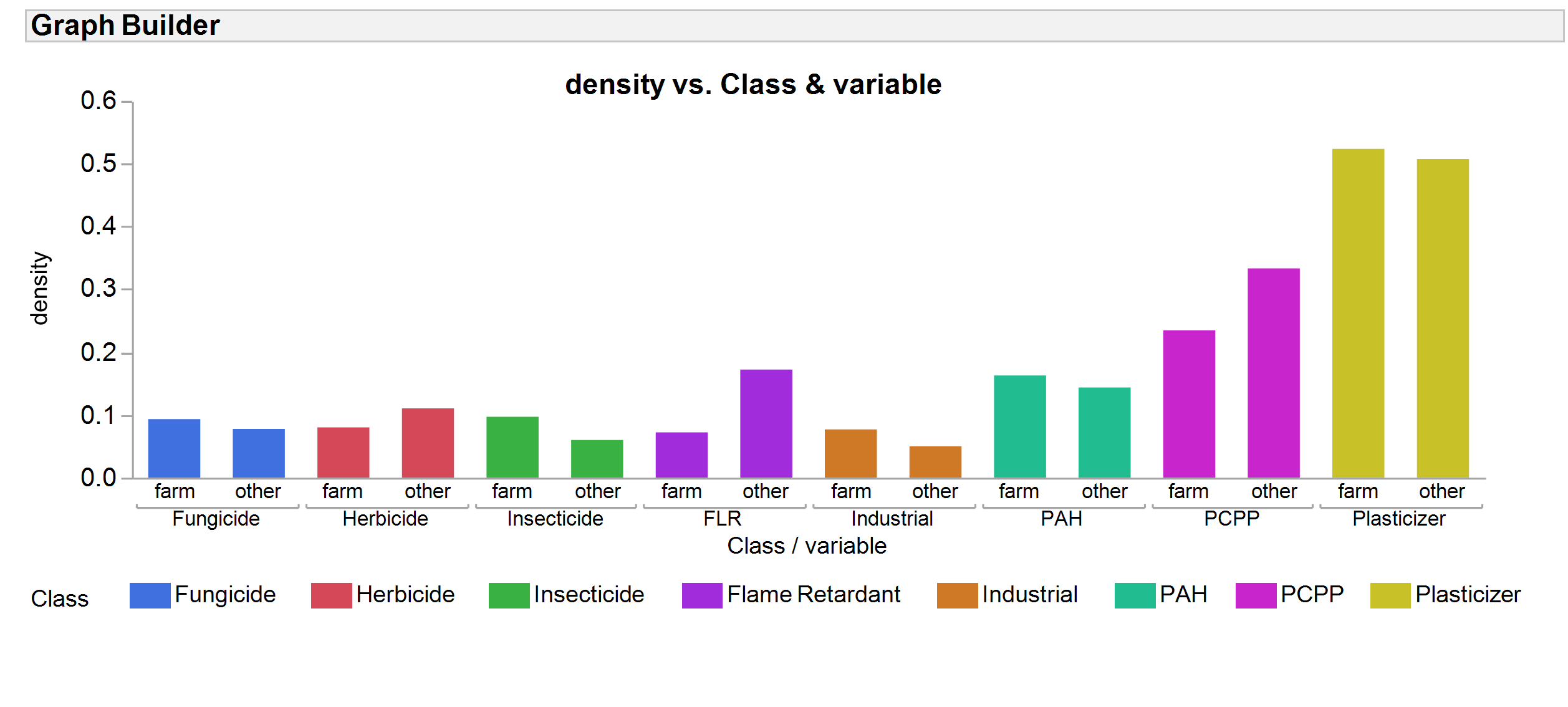

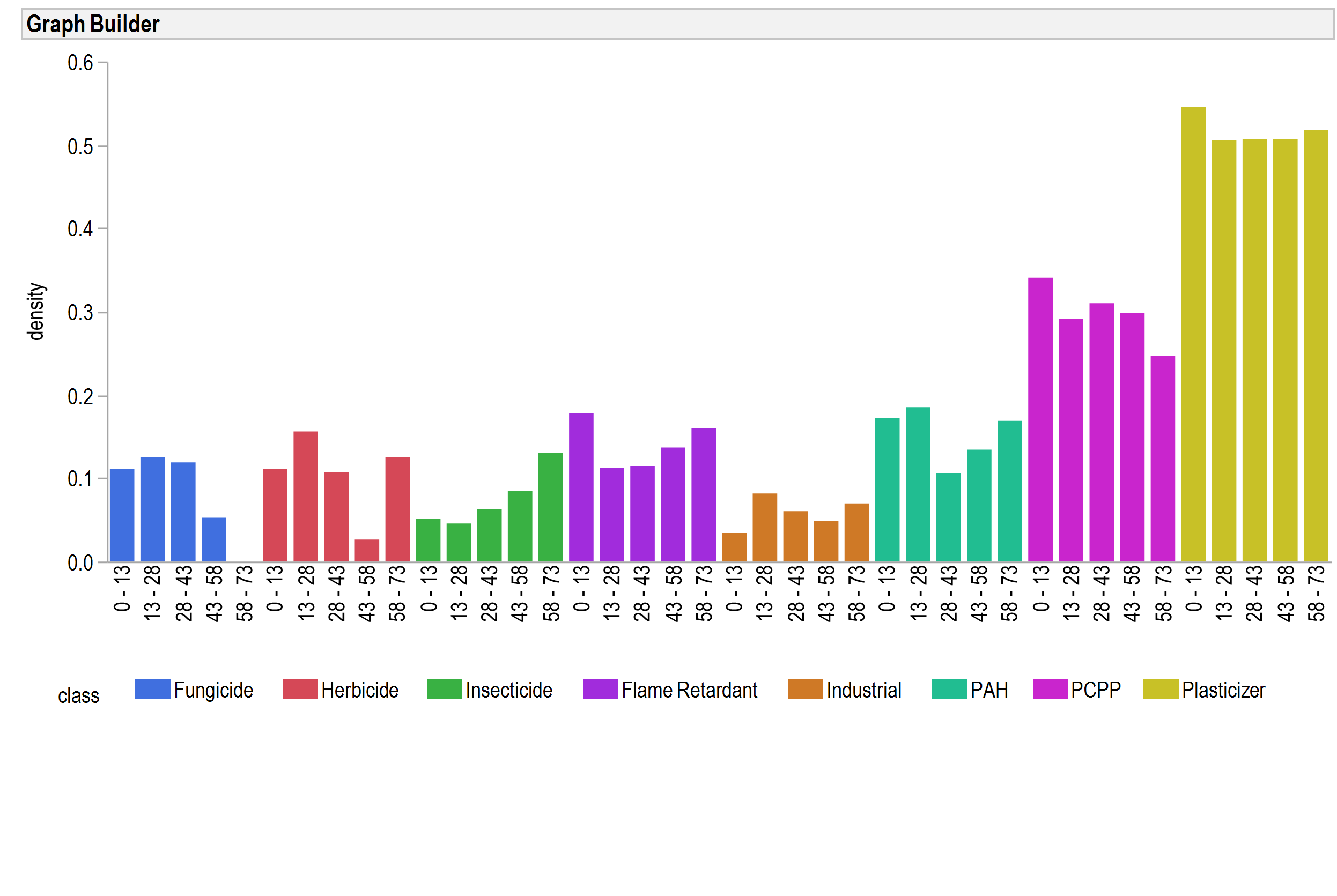


Proportion detected

Age

p = 0.9714

p = 0.4069

p = 0.8191

p = 0.5116

p = 0.0075

**p = 0.0007**

p = 0.0719

p = 0.0971

Figure S6. Effect of Age on the detection rate of eight chemical classes in wristbands worn by residents of the Alto Mayo. Age groups were compared with a Chi-squared likelihood ratio test.

| Table S4. Statistical results of the Chi-square likelihood ratio test comparing community for the *ln*(concentration + 1) of the top three pesticides as seen in Figure 4. | | | | | | | |
| --- | --- | --- | --- | --- | --- | --- | --- |
| **Compound** | **Community 1** | **vs** | **Community 2** | **Score Mean Difference** | **Standard Error** | **Z** | **p-Value** |
| chlorpyrifos | Rioja | vs | Yantaló | 6.35 | 3.80 | 1.67 | 0.095 |
|  | Rioja |  | Tingana | 4.52 | 3.06 | 1.48 | 0.139 |
|  | Tingana |  | Yantaló | 2.69 | 3.80 | 0.71 | 0.479 |
|  | Moyobamba |  | Yantaló | -9.47 | 3.79 | -2.50 | 0.013 |
|  | Moyobamba |  | Tingana | -9.54 | 3.00 | -3.18 | 0.002 |
|  | Moyobamba |  | Rioja | -9.64 | 3.05 | -3.16 | 0.002 |
|  |  |  |  |  |  |  |  |
| cypermethrin | Moyobamba |  | Rioja | 0.00 | 2.88 | 0.00 | 1.000 |
|  | Moyobamba |  | Tingana | -2.31 | 2.94 | -0.79 | 0.432 |
|  | Rioja |  | Tingana | -3.56 | 3.02 | -1.18 | 0.238 |
|  | Tingana |  | Yantaló | -6.49 | 3.79 | -1.71 | 0.087 |
|  | Moyobamba |  | Yantaló | -8.54 | 3.75 | -2.27 | 0.023 |
|  | Rioja |  | Yantaló | -9.25 | 3.77 | -2.45 | 0.014 |
|  |  |  |  |  |  |  |  |
| DDx | Moyobamba |  | Rioja | 2.15 | 3.06 | 0.70 | 0.482 |
|  | Tingana |  | Yantaló | -0.12 | 3.80 | -0.03 | 0.976 |
|  | Moyobamba |  | Tingana | -7.08 | 3.00 | -2.36 | 0.018 |
|  | Rioja |  | Tingana | -9.12 | 3.06 | -2.99 | 0.003 |
|  | Moyobamba |  | Yantaló | -9.35 | 3.80 | -2.46 | 0.014 |
|  | Rioja |  | Yantaló | -11.76 | 3.81 | -3.09 | 0.002 |

Table S5. Alternative multiple linear regression results. Non-detects substituted with LOD/sqrt(2).

|  | **Parameter** | **Estimate** | **SE** | **p** |  | **r^2^** |
| --- | --- | --- | --- | --- | --- | --- |
| *ln*(chlorpyrifos) | Intercept | 5.29 | 0.24 | **<0.0001** |  | 0.20 |
|  | *Community* |  |  |  |  |  |
|  | Yantaló-Moyobamba | 0.00 | 0.35 | 0.9967 |  |  |
|  | Tingana-Moyobamba | 0.43 | 0.43 | 0.3249 |  |  |
|  | Rioja-Moyobamba | 1.11 | 0.42 | **0.0098** |  |  |
|  |  |  |  |  |  |  |
| *ln*(cypermethrin) | Intercept | 3.02 | 0.66 | **<0.0001** |  | 0.20 |
|  | *Community* |  |  |  |  |  |
|  | Yantaló-Moyobamba | 1.08 | 0.44 | **0.0164** |  |  |
|  | Tingana-Moyobamba | 0.31 | 0.54 | 0.5740 |  |  |
|  | Rioja-Moyobamba | -0.58 | 0.52 | 0.2696 |  |  |
|  | Age | 0.03 | 0.02 | **0.0418** |  |  |
|  |  |  |  |  |  |  |
| *ln*(DDx) | Intercept | 4.17 | 0.34 | **<0.0001** |  | 0.40 |
|  | *Community* |  |  |  |  |  |
|  | Yantaló-Moyobamba | 0.66 | 0.22 | **0.0041** |  |  |
|  | Tingana-Moyobamba | 0.42 | 0.28 | 0.1372 |  |  |
|  | Rioja-Moyobamba | -0.76 | 0.26 | **0.0053** |  |  |
|  | Gender (male) | 0.20 | 0.15 | 0.1836 |  |  |
|  | Age | 0.03 | 0.01 | **0.0025** |  |  |

References Cited

1. Anderson KA, Sethajintanin D, Sower G, Quarles L. Field trial and modeling of uptake rates of *in situ* lipid-free polyethylene membrane passive sampler. Environ Sci Technol. 2008;42:4486-93.

2. O'Connell SG, McCartney MA, Paulik LB, Allan SE, Tidwell LG, Wilson G, et al. Improvements in pollutant monitoring: optimizing silicone for co-deployment with polyethylene passive sampling devices. Environ Pollut. 2014;193:71-8.

3. Anderson KA, Seck D, Hobbie KA, Traore AN, McCartney MA, Ndaye A, et al. Passive sampling devices enable capacity building and characterization of bioavailable pesticide along the Niger, Senegal and Bani Rivers of Africa. Philos Trans R Soc B: Biol Sci. 2014;369:20130110.

4. Donald CE, Scott RP, Blaustein KL, Halbleib ML, Sarr M, Jepson PC, et al. Silicone wristbands detect individuals' pesticide exposures in West Africa. R Soc Open Sci. 2016;3(8):160433.

(2,3-Dibromopropyl) (2,4,6-tribromophenyl) ether

1,2,3,4,6,7,8,9-Octachlorodibenzofuran

1,2,3,4,6,7,8,9-Octachlorodibenzo-p-dioxin

1,2,3,4,6,7,8-Heptachlorodibenzofuran

1,2,3,4,6,7,8-Heptachlorodibenzo-p-dioxin

1,2,3,4,6,7,9-Heptachlorodibenzo-p-dioxin

1,2,3,4,6,7-Hexachlorodibenzo-p-dioxin

1,2,3,4,7,8-Hexachlorodibenzofuran

1,2,3,4,7,8-Hexachlorodibenzo-p-dioxin

1,2,3,4,7-Pentachlorodibenzo-p-dioxin

1,2,3,4-Tetrachlorodibenzofuran

1,2,3,4-Tetrachlorodibenzo-p-dioxin

1,2,3,6,7,8-Hexachlorodibenzo-p-dioxin

1,2,3,7,8,9-Hexachlorodibenzo-p-dioxin

1,2,3,7,8-Pentachlorodibenzofuran

1,2,3,7,8-Pentachlorodibenzo-p-dioxin

1,2,3,8,9-Pentachlorodibenzo-p-dioxin

1,2,3-Trichlorodibenzo-p-dioxin

1,2,4,6,7,9/1,2,4,6,8,9-Hexachlorodibenzo-p-dioxin

1,2,4,6,8/1,2,4,7,9-Pentachlorodibenzo-p-dioxin

1,2,4,7,8-Pentachlorodibenzo-p-dioxin

1,2,4-Trichlorobenzene

1,2,4-Trichlorodibenzo-p-dioxin

1,2,5,6,9,10-Hexabromocyclododecane

1,2,6,7-Tetrachlorodibenzo-p-dioxin

1,2,6,8-Tetrachlorodibenzo-p-dioxin

1,2,7,8-Tetrachlorodibenzo-p-dioxin

1,2,8,9-Tetrachlorodibenzo-p-dioxin

1,2-Bis(2,4,6-tribromophenoxy)ethane

1,2-Dibromo-3-chloropropane

1,2-Dibromo-4-(1,2-dibromoethyl)cyclohexane

1,2-Dichlorodibenzo-p-dioxin

1,2-dimethylnaphthalene

1,3,5-Tribromobenzene

1,3,6,8-Tetrachlorodibenzofuran

1,3,6,8-Tetrachlorodibenzo-p-dioxin

1,3,7,8-Tetrachlorodibenzo-p-dioxin

1,3,7,9-Tetrachlorodibenzo-p-dioxin

1,3-Dichlorobenzene

1,3-Dichlorodibenzo-p-dioxin

1,3-Dinitropyrene

1,4-Anthraquinone

1,4-Dichlorodibenzo-p-dioxin

1,4-dimethylnaphthalene

1,4-Dioxino(2,3,b,5,6,b')dipyridine

1,5-dimethylnaphthalene

1,6-Benzo(a)pyrene-quinone

1,6-Dichlorodibenzo-p-dioxin

1,6-dimethylnaphthalene

1,6-Dinitropyrene

1,7,8-Trichlorodibenzo-p-dioxin

1,8-dimethylnaphthalene

1,8-Dinitropyrene

17a-Ethynylestradiol

1-Chlorodibenzo-p-dioxin

1-Hydroxynaphthalene

1-methylnaphthalene

1-methylphenanthrene

1-methylpyrene

1-Nitronaphthalene

1-Nitropyrene

2-(1-naphthyl)acetamide

2-(2-Butoxyethoxy)ethyl thiocyanate

2-(3-Chlorophenoxy)propionamide

2-(Octylthio)ethanol

2,2',3,3',4,4'-Hexabromodiphenyl ether

2,2',3,3',4,5',6,6'-Octabromodiphenyl ether

2,2',3,4,5,5',6-Heptabromodiphenyl ether

2,2',3,4,5,6-Hexabromodiphenyl ether

2,2',3,4,5',6-Hexabromodiphenyl ether

2,2',3,4,6'-Pentabromodiphenyl ether

2,2',3,4,6-Pentabromodiphenyl ether

2,2',4,5',6-Pentabromodiphenyl ether

2,2',4,6'-Tetrabromodiphenyl ether

2,2',4,6-Tetrabromodiphenyl ether

2,2',5-Tribromobiphenyl

2,2',6-Tribromodiphenyl ether

2,2'-Dibromobiphenyl

2,2'-Dibromodiphenyl ether

2,3,3',4,5,6-Hexabromodiphenyl ether

2,3,3',4,5'-Pentabromodiphenyl ether

2,3,4,4',6-Pentabromodiphenyl ether

2,3,4,5-Tertrachloronitrobenzene

2,3,4,5-Tetrachlorophenol

2,3,4,6-Tetrabromodiphenyl ether

2,3',4,6-Tetrabromodiphenyl ether

2,3,4,6-Tetrachlorophenol

2,3,4,7,8-PeCDF

2,3,4-Tribromodiphenyl ether

2,3,4-Tribromophenol

2,3,4-Trichlorophenyl-4-nitrophenyl ether

2,3,5,6-Tetrachlorophenol

2,3,5,6-Tetrachloro-p-terphenyl

2,3',5-Tribromobiphenyl

2,3',5-Tribromodiphenyl ether

2,3,5-Trichlorophenol

2,3,5-Trichlorophenyl-4-nitrophenyl ether

2,3,5-Trimethacarb

2,3',6-Tribromodiphenyl ether

2,3,6-Trichloroanisole

2,3,6-Trichlorophenyl-4-nitrophenyl ether

2,3,7,8-TCDD

2,3,7,8-Tetrabromodibenzo-p-dioxin

2,3,7,8-Tetrachlorodibenzofuran

2,3,7-Trichlorodibenzo-p-dioxin

2,3-Dibromoanisole

2,3'-Dibromodiphenyl ether

2,3-Dibromophenol

2,3-Dichlorodibenzo-p-dioxin

2,3-Dichlorophenyl-4-nitrophenyl ether

2,3-dimethylanthracene

2,4,5-T methyl ester

2,4,5-Tribromoanisole

2,4',5-Tribromodiphenyl ether

2,4,5-Trichloroaniline

2,4,5-Trichlorophenol

2,4,5-Trichlorophenyl-4-nitrophenyl ether

2,4,5-Trichloro-p-terphenyl

2,4,5-Trimethylaniline

2,4,6-Tribromoanisole

2,4,6-Tribromophenol

2,4,6-Tribromophenyl allyl ether

2,4,6-Trichloroanisole

2,4,6-Trichlorophenol

2,4,8-Trichlorodibenzofuran

2,4-D methyl ester

2,4-D sec-butyl ester

2,4-DB methyl ester

2,4'-DDD

2,4'-DDE

2,4'-DDT

2,4-Dibromoanisole

2,4-Dibromobiphenyl

2,4-Dibromophenol

2,4-Dibromophenyl-4-nitrophenyl ether

2,4'-Dichlorobenzophenone (2,4'-Dicofol decomposition product)

2,4-Dichlorophenol

2,4-Dichlorophenyl benzenesulfonate

2,4-Dimethylaniline

2,4-Dimethylphenol

2,5-Dibromoanisole

2,5-Dibromobiphenyl

2,5-Dibromodiphenyl ether

2,5-Dibromophenol

2,5-Dichlorophenyl-4-nitrophenyl ether

2,6-Dibromoanisole

2,6-Dibromobiphenyl

2,6-Dibromophenol

2,6-Dichlorobenzamide

2,6-Dichlorobenzonitrile

2,6-Dichlorophenol

2,6-Dichlorophenyl-4-nitrophenyl ether

2,6-Dichlorosyringaldehyde

2,6-diethylnaphthalene

2,6-Dimethylaniline

2,6-dimethylnaphthalene

2,7-Dichlorodibenzo-p-dioxin

2,8-Dichlorodibenzofuran

2,8-Dichlorodibenzo-p-dioxin

2-Bromoanisole

2-Bromobiphenyl

2-chlorodibenzofuran

2-Chlorodibenzo-p-dioxin

2-Chlorophenol

2-Chlorophenyl-4-nitrophenyl ether

2-Chlorosyringaldehyde

2-Ethyl-1,3-hexanediol

2-ethyl-6-methylaniline

2-ethylnaphthalene

2'-Hydroxy-2,4,4'-tribromodiphenyl ether

2'-Hydroxy-4-monobromodiphenyl ether

2-Hydroxyestradiol

2'-Methoxy-2,4,4'-tribromodiphenyl ether

2-methylanthracene

2-methylnaphthalene

2-methylphenanthrene

2-Methylphenol

2-Nitroanthracene

2-Nitrobiphenyl

2-Nitrofluorene

2-Nitronaphthalene

2-Nitrophenol

2-Nitropyrene

2-Phenoxypropionic acid

3,3',4,5,5'-Pentabromodiphenyl ether

3,3'-Dimethoxybenzidine

3,4,5-Trichlorophenyl-4-nitrophenyl ether

3,4,5-Trimethacarb

3,4,6-Trichloroguaiacol

3,4-Dichloroaniline

3,4-Dichlorocatechol

3,4-Dichloroguaiacol

3,4-Dichlorophenyl-4-nitrophenyl ether

3,5-Dibromoanisole

3,5-Dibromophenol

3,5-Dichloroaniline

3,5-Dichlorophenyl-4-nitrophenyl ether

3,6-dimethylphenanthrene

3-Aminophenol

3-Bromoanisole

3-Bromobiphenyl

3-Bromophenol

3-Bromostyrene

3-Chloro-4-fluoroaniline

3-Chloro-4-methoxyaniline

3-Chloroaniline

3-Chlorophenyl-4-nitrophenyl ether

3-Hydroxycarbofuran

3-Indolylacetonitrile

3-Methoxy-2,2',4,4',6-pentabromodiphenyl ether

3-Nitrobenzanthrone

3-Nitrobiphenyl

3-nitrodibenzofuran

3-Nitrofluoranthene

3-Nitrophenanthrene

3-Trifluormethylaniline

4,4'-DDD

4,4'-DDE

4,4'-DDT

4,4'-Dibromobiphenyl

4,4'-Dichlorobenzophenone

4,4'-Oxydianiline

4,5-Dichloroguaiacol

4,6-Dichloroguaiacol

4,6-Dinitro-o-cresol (DNOC)

4-Aminobiphenyl

4-Bromoaniline

4-Bromoanisole

4-Bromobiphenyl

4-Bromophenol

4-Bromostyrene

4-Chloro-2-methylaniline

4-Chloro-3-methylphenol

4-Chloroaniline

4-Chlorocatechol

4-Chlorodibenzofuran

4-Chloroguaiacol

4-Chlorophenol

4-Chlorophenyl isocyanate

4-Chlorophenyl phenyl ether

4-Chlorophenyl-4-nitrophenyl ether

4H-Cyclopenta(def)phenanthren-4-one

4-Isopropylaniline

4-Methylphenol

4-Nitrobiphenyl

4-Nitrophenol

4-Nitrophenyl phenyl ether

4-Nonylphenol

5,12-Naphthacene-quinone

5,6-Dichlorovanillin

5,7-Dihydroxy-4'-methoxyisoflavone

5-Chlorovanillin

5-methylchrysene

5-Nitroacenaphthene

6-Chlorovanillin

6-methylchrysene

6-Nitrobenzo(a)pyrene

6-Nitrochrysene

7,12-dimethylbenz[a]anthracene

7-Nitrobenz(a)anthracene

9,10-Anthraquinone

9,10-dimethylanthracene

9,10-Phenanthrenequinone

9-Fluorenone

9-methylanthracene

9-Nitroanthracene

9-Nitrophenanthrene

acenaphthene

Acenaphthenequinone

acenaphthylene

Acephate

Acequinocyl

acetamiprid

Acetochlor

Acifluorfen methyl ester

Aclonifen

Acrinathrin

Alachlor

Aldrin

Allidochlor

alpha, alpha-Dibromo-m-xylene

alpha-BHC

alpha-Chlordane

Ametryne

Amidithion

Aminocarb

Amitraz

Amitraz metabolite [Methanimidamide, N-(2,4-dimethylphenyl)-N'-methyl-]

Ancymidol

Anilazine

Aniline

Anilofos

anthanthrene

anthracene

Aramite

Aramite II

Atraton

Atrazine

Atrazine-desethyl

Azaconazole

Azamethiphos

Azibenzolar-S-methyl

Azinphos-ethyl

Azinphos-methyl

Aziprotryn metabolite [2-Amino-4-isopropylamino-6-methylthio-1,3,5-triazine]

Aziprotryne

Azobenzene

Azoxybenzene

Azoxystrobin

Barban

Beflubutamid

Benalaxyl

Benazolin-ethyl

Bendiocarb

Benfluralin

Benfuracarb

Benfuresate

Benodanil

Benoxacor

Bentazone

Bentazone methyl derivative

Benthiocarb

Benz(a)anthracene-7,12-dione

benz[a]anthracene

benz[j]and[e]aceanthrylene

Benzanthrone

Benzenesulfonamide

Benzidine

Benzo(a)fluoren-11-one

Benzo(a)pyrene-7,8-dione

Benzo(c)phenanthrene(1,4)quinone

benzo[a]chrysene

benzo[a]fluorene

benzo[a]pyrene

benzo[b]fluoranthene

benzo[b]fluorene

benzo[b]perylene

benzo[c]fluorene

benzo[e]pyrene

benzo[ghi]perylene

benzo[j]fluoranthene

benzo[k]fluoranthene

Benzophenone

Benzoximate metabolite

Benzoylprop ethyl

Benzyl benzoate

b-Estradiol

beta-BHC

BHC epsilon isomer

Bifenazate metabolite (5-Phenyl-o-anisidine )

Bifenox

Bifenthrin

Binapacryl

Bioallethrin

Bioallethrin S-cyclopentenyl isomer

Bioresmethrin

Biphenyl

Bis(2,3,3,3-tetrachloropropyl) ether

Bis(2-butoxyethyl) phthalate

Bis(2-ethylhexyl)phthalate

Bisphenol A

Bitertanol I

Bitertanol II

Boscalid (Nicobifen)

Bromacil

Bromfenvinphos-(E)

Bromfenvinphos-(Z)

Bromobutide

Bromocyclen

Bromophos

Bromophos-ethyl

Bromopropylate

Bromoxynil

Bromoxynil octanoic acid ester

Bromuconazole I

Bromuconazole II

Bufencarb

Bupirimate

Buprofezin

Butachlor

Butafenacil

Butamifos

Butoxycarboxim

Butralin

Butyl benzyl phthalate

Butylate

Butylated hydroxyanisole

Cadusafos

Cafenstrole

Caffeine

Captafol

Captan

Carbaryl

Carbetamide

Carbofuran

Carbofuran-3-keto

Carbofuran-7-phenol

Carbophenothion

Carbosulfan

Carboxin

Carfentrazone-ethyl

Carpropamid

Carvone

Cashmeran

Cekafix

Celestolide

Chinomethionat

Chloramben methyl ester

Chloranocryl

Chlorbenside

Chlorbenside sulfone

Chlorbicyclen

Chlorbromuron

Chlorbufam

Chlordene, trans-

Chlordimeform

Chlorethoxyfos

Chlorfenapyr

Chlorfenethol

Chlorfenprop-methyl

Chlorfenson

Chlorfenvinphos

Chlorfenvinphos, cis-

Chlorfenvinphos, trans-

Chlorflurecol-methyl ester

Chlormefos

Chlornitrofen

Chlorobenzilate

Chloroneb

Chloropropylate

Chlorothalonil

Chlorotoluron

Chlorpropham

Chlorpyrifos

Chlorpyrifos Methyl

Chlorthiamid

Chlorthion

Chlorthiophos

Chlorthiophos sulfone

Chlorthiophos sulfoxide

Chlozolinate

chrysene

Cinerin I

Cinerin II

Cinidon-ethyl

cis-Nonachlor

Clodinafop-propargyl

Clomazone

Cloquintocet-mexyl

coronene

Coumaphos

Crimidine

Crotoxyphos

Crufomate

Cyanazine

Cyanofenphos

Cyanophos

Cyclafuramid

Cycloate

cyclopenta[cd]pyrene

Cyclopentadecanone

Cycluron

Cyflufenamid

Cyfluthrin I

Cyfluthrin II

Cyfluthrin III

Cyfluthrin IV

Cyhalofop-butyl

Cyhalothrin (Gamma)

Cyhalothrin I (lambda)

Cymiazole

Cymoxanil

Cypermethrin-1

Cypermethrin-2

Cypermethrin-3

Cypermethrin-4

Cyphenothrin cis-

Cyphenothrin trans-

Cyprazine

Cyproconazole

Cyprodinil

Cyprofuram

Cyromazine

d-(cis-trans)-Phenothrin-I

d-(cis-trans)-Phenothrin-II

Dacthal

Dazomet

DDMU [1-Chloro-2,2-bis(4'-chlorophenyl)ethylene]

delta-BHC

Deltamethrin

Demephion

Demeton-s

Demeton-S-methyl

Demeton-S-methylsulfon

Desbromo-bromobutide

Desmedipham

Desmetryn

Dialifos

Diallate I

Diallate II

Diamyl phthalate

Diazinon

Diazinon-oxon

dibenzo[a,e]fluoranthene

dibenzo[a,e]pyrene

dibenzo[a,h]anthracene

dibenzo[a,h]pyrene

dibenzo[a,i]pyrene

dibenzo[a,l]pyrene

dibenzo[e,l]pyrene

Dibenzofuran

Dibenzo-p-dioxin

dibenzothiophene

Dicamba

Dicamba methyl ester

Dicapthon

Dichlofenthion

Dichlofluanid

Dichlofluanid metabolite (DMSA)

Dichlone

Dichloran

Dichlormid

Dichlorophen

Dichlorprop

Dichlorprop methyl ester

Dichlorvos

Diclobutrazol

Diclocymet I

Diclocymet II

Diclofop methyl

Dicrotophos

Dicyclohexyl phthalate

Dicyclopentadiene

Dieldrin

Diethatyl ethyl

Diethofencarb

Diethyl dithiobis(thionoformate) (EXD)

Diethyl phthalate

Diethylene glycol

Diethylstilbestrol

Difenoconazol I

Difenoconazol II

Difenoxuron

Diflufenican

Diisobutyl phthalate

Dimefox

Dimepiperate

Dimethachlor

Dimethametryn

Dimethenamid

Dimethipin

Dimethoate

Dimethomorph-(E)

Dimethomorph-(Z)

Dimethyl phthalate

Dimethylvinphos(E)

Dimethylvinphos(Z)

Dimetilan

Dimoxystrobin

Di-n-butyl phthalate

Di-n-hexyl phthalate

Diniconazole

Dinitramine

Di-n-nonyl phthalate

Dinobuton

Dinocap

Dinocap II

Dinocap III

Dinocap IV

Di-n-octyl phthalate

Dinoseb

Dinoseb acetate

Dinoseb methyl ether

Dinoterb

Dinoterb acetate

Di-n-propyl phthalate

Diofenolan I

Diofenolan II

Dioxabenzofos

Dioxacarb

Dioxathion

Diphacinone

Diphenamid

Diphenyl phthalate

Diphenylamine

Dipropetryn

Dipropyl isocinchomeronate

Disulfoton

Disulfoton sulfone

Ditalimfos

Dithiopyr

Diuron

Diuron Metabolite [3,4-Dichlorophenyl isocyanate]

Dodemorph I

Dodemorph II

Drazoxolon

Edifenphos

Empenthrin I

Empenthrin II

Empenthrin III

Empenthrin IV

Empenthrin V

Endosulfan ether

Endosulfan I

Endosulfan II

Endosulfan lactone

Endosulfan sulfate

Endrin

Endrin aldehyde

Endrin ketone

EPN

Epoxiconazole

EPTC

Erbon

Esfenvalerate

Esprocarb

Etaconazole

Ethalfluralin

Ethidimuron

Ethiofencarb

Ethiolate

Ethion

Ethofenprox

Ethofumesate

Ethofumesate, 2-Keto

Ethoprophos

Ethoxyfen-ethyl

Ethoxyquin

Ethylenethiourea

Etoxazole

Etridiazole, deschloro- (5-ethoxy-3-dichloromethyl-1,2,4-thiadiazole)

Etrimfos

Eugenol

Exaltolide [15-Pentadecanolide]

Famoxadon

Famphur

Fenamidone

Fenamiphos

Fenamiphos sulfoxide

Fenamiphos-sulfone

Fenarimol

Fenazaflor

Fenazaflor metabolite

Fenazaquin

Fenbuconazole

Fenchlorazole-ethyl

Fenchlorphos

Fenchlorphos-oxon

Fenclorim

Fenfuram

Fenhexamid

Fenitrothion

Fenitrothion-oxon

Fenobucarb

Fenoprop

Fenoprop methyl ester

Fenothiocarb

Fenoxanil

Fenoxaprop-ethyl

Fenoxycarb

Fenpiclonil

Fenpropathrin

Fenpropidin

Fenson

Fensulfothion

Fensulfothion-oxon

Fensulfothion-oxon -sulfone

fensulfothion-sulfone

Fenthion

Fenthion sulfoxide

Fenthion-sulfone

Fenuron

Fenvalerate

Fenvalerate II

Fepropimorph

Fipronil

Fipronil, Desulfinyl-

Fipronil-sulfide

Fipronil-sulfone

Flamprop-isopropyl

Flamprop-methyl

Fluacrypyrim

Fluazifop-p-butyl

Fluazinam

Fluazolate

Flubenzimine

Fluchloralin

Flucythrinate I

Flucythrinate II

Fludioxonil

Flufenacet

Flumetralin

Flumiclorac-pentyl

Flumioxazin

Fluometuron

fluoranthene

fluorene

Fluorodifen

Fluoroglycofen-ethyl

Fluoroimide

Fluotrimazole

Fluoxastrobin cis-

Fluquinconazole

Flurenol-butyl ester

Flurenol-methylester

Fluridone

Flurochloridone I

Flurochloridone II

Flurochloridone, deschloro-

Fluroxypyr-1-methylheptyl ester

Flurprimidol

Flurtamone

Flusilazole

Fluthiacet-methyl

Flutolanil

Flutriafol

Fluvalinate-tau-I

Fluvalinate-tau-II

Folpet

Fonofos

Formothion

Fosthiazate I

Fosthiazate II

Fuberidazole

Furalaxyl

Furathiocarb

Furilazole

Furmecyclox

Galaxolide

gamma-Chlordane

Halfenprox

Haloxyfop-methyl

Heptachlor

Heptachlor epoxide

Heptachlor epoxide isomer A

Heptenophos

Hexabromobenzene

Hexachlorobenzene

Hexachlorophene

Hexaconazole

Hexazinone

Hexestrol

Hydroprene

Imazalil

Imazamethabenz-methyl I

Imazamethabenz-methyl II

Imibenconazole

Imibenconazole-desbenzyl

Imidan

indeno[1,2,3-cd]pyrene

Indoxacarb and Dioxacarb decomposition product [Phenol, 2-(1,3-dioxolan-2-yl)-]

Ioxynil

Ioxynil octanoate

Ipconazole

Iprobenfos

Iprodione

Iprovalicarb I

Iprovalicarb II

Irgarol

Isazophos

Isobenzan

Isobornyl thiocyanoacetate

Isocarbamide

Isocarbophos

Isodrin

Isofenphos

Isofenphos-oxon

Isomethiozin

Isoprocarb

Isopropalin

Isoprothiolane

Isoproturon

Isoxaben

Isoxadifen-ethyl

Isoxaflutole

Isoxathion

Jasmolin I

Jasmolin II

Jodfenphos

Kepone

Kinoprene

Kresoxim-methyl

Lactofen

Lenacil

Leptophos

Leptophos oxon

Lindane

Linuron

Malathion

Malathion-o-analog

MCPA methyl ester

MCPA-butoxyethyl ester

MCPB methyl ester

m-Cresol

Mecarbam

Mecoprop methyl ester

Mefenacet

Mefenpyr-diethyl

Mefluidide

Menazon

Mepanipyrim

Mephosfolan

Mepronil

Metalaxyl

Metamitron

Metazachlor

Metconazole I

Metconazole II

Methabenzthiazuron [decomposition product]

Methacrifos

Methamidophos

Methfuroxam

Methidathion

Methiocarb

Methiocarb Sulfone

Methiocarb sulfoxide

Methomyl

Methoprene I

Methoprene II

Methoprotryne

Methoxychlor

Methoxychlor olefin

Methyl (2-naphthoxy)acetate

Methyl paraoxon

Methyl-1-naphthalene acetate

Methyldymron

Metobromuron

Metolachlor

Metolcarb

Metominostrobin (E)

Metominostrobin (Z)

Metrafenone

Metribuzin

Mevinphos

Mirex

Molinate

Monalide

Monocrotophos

Monolinuron

Musk amberette

Musk Ketone

Musk Moskene

Musk Tibetene (Moschustibeten)

Musk xylene

Myclobutanil

N,N-Diethyl-m-toluamide

N-1-Naphthylacetamide

Naled

naphthalene

Naphthalic anhydride

Naphthanthrone

naphtho[1,2-b]fluoranthene

naphtho[2,3-a]pyrene

naphtho[2,3-e]pyrene

naphtho[2,3-j]fluoranthene

naphtho[2,3-k]fluoranthene

Naproanilide

Napropamide

Nickel dibutyldithiocarbamate

Nicotine

Nitralin

Nitrapyrin

Nitrofen

Nitrothal-isopropyl

N-Methyl-N-1-naphthyl acetamide

Norflurazon

Norflurazon, Desmethyl-

Nuarimol

Octachlorostyrene

Octamethyl pyrophosphoramide

o-Dichlorobenzene

Ofurace

Omethoate

o-Phenylphenol

Orbencarb

ortho-Aminoazotoluene

Oryzalin

Oxabetrinil

Oxadiazon

Oxadixyl

Oxamyl

Oxycarboxin

Oxychlordane

Oxydemeton-methyl

Oxyfluorfen

p,p'-DDM [bis(4-chlorophenyl)methane]

p,p'-Dibromobenzophenone

p,p'-Dicofol

Paclobutrazol

Paraoxon

Parathion-ethyl

Parathion-methyl

PBB 101

PBB 169 Hexabrombiphenyl

PBB 52 Tetrabrombiphenyl

PBB-101

PBB-103

PBB-114

PBB-137

PBB-141

PBB-153

PBB-155

PBB-156

PBB-159

PBB-169

PBB-180

PBB-189

PBB-200

PBB-29

PBB-30

PBB-31

PBB-49

PBB-53

PBB-77

PBB-80

PBDE 1

PBDE 10

PBDE 100

PBDE 11

PBDE 116

PBDE 118

PBDE 119

PBDE 12

PBDE 13

PBDE 138

PBDE 15

PBDE 153

PBDE 154

PBDE 155

PBDE 166

PBDE 17

PBDE 2

PBDE 25

PBDE 28

PBDE 3

PBDE 30

PBDE 32

PBDE 33

PBDE 35

PBDE 37

PBDE 47

PBDE 49

PBDE 66

PBDE 7

PBDE 71

PBDE 75

PBDE 77

PBDE 8

PBDE 85

PBDE 99

PCB 1

PCB 10

PCB 101

PCB 102

PCB 103

PCB 104

PCB 105

PCB 106

PCB 107

PCB 108

PCB 109

PCB 11

PCB 110

PCB 111

PCB 112

PCB 113

PCB 114

PCB 115

PCB 116

PCB 117

PCB 118

PCB 119

PCB 12

PCB 120

PCB 121

PCB 122

PCB 123

PCB 124

PCB 125

PCB 126

PCB 127

PCB 128

PCB 129

PCB 13

PCB 130

PCB 131

PCB 132

PCB 133

PCB 134

PCB 135

PCB 136

PCB 137

PCB 138

PCB 139

PCB 14

PCB 140

PCB 141

PCB 142

PCB 143

PCB 144

PCB 145

PCB 146

PCB 147

PCB 148

PCB 149

PCB 15

PCB 150

PCB 151

PCB 152

PCB 153

PCB 154

PCB 155

PCB 156

PCB 157

PCB 158

PCB 159

PCB 16

PCB 160

PCB 161

PCB 162

PCB 163

PCB 164

PCB 165

PCB 166

PCB 167

PCB 168

PCB 169

PCB 17

PCB 170

PCB 171

PCB 172

PCB 173

PCB 174

PCB 175

PCB 176

PCB 177

PCB 178

PCB 179

PCB 18

PCB 180

PCB 181

PCB 182

PCB 183

PCB 184

PCB 185

PCB 186

PCB 187

PCB 188

PCB 189

PCB 19

PCB 190

PCB 191

PCB 192

PCB 193

PCB 194

PCB 195

PCB 196

PCB 197

PCB 198

PCB 199

PCB 2

PCB 20

PCB 200

PCB 201

PCB 202

PCB 203

PCB 204

PCB 205

PCB 206

PCB 207

PCB 208

PCB 21

PCB 22

PCB 23

PCB 24

PCB 25

PCB 26

PCB 27

PCB 28

PCB 29

PCB 3

PCB 30

PCB 31

PCB 32

PCB 33

PCB 34

PCB 35

PCB 36

PCB 37

PCB 38

PCB 39

PCB 4

PCB 40

PCB 41

PCB 42

PCB 43

PCB 44

PCB 45

PCB 46

PCB 47

PCB 48

PCB 49

PCB 5

PCB 50

PCB 51

PCB 52

PCB 53

PCB 54

PCB 55

PCB 56

PCB 57

PCB 58

PCB 59

PCB 6

PCB 60

PCB 61

PCB 62

PCB 63

PCB 64

PCB 65

PCB 66

PCB 67

PCB 68

PCB 69

PCB 7

PCB 70

PCB 71

PCB 72

PCB 73

PCB 74

PCB 75

PCB 76

PCB 77

PCB 78

PCB 79

PCB 8

PCB 80

PCB 81

PCB 82

PCB 83

PCB 84

PCB 85

PCB 86

PCB 87

PCB 88

PCB 89

PCB 9

PCB 90

PCB 91

PCB 92

PCB 93

PCB 94

PCB 95

PCB 96

PCB 97

PCB 98

PCB 99

p-Dichlorobenzene

Pebulate

Penconazole

Pendimethalin

Pentabromoethylbenzene

Pentabromotoluene

Pentachloroaniline

Pentachloroanisole

Pentachlorobenzene

Pentachloronitrobenzene

Pentachlorophenol

Pentanochlor

Permethrin

Permethrin II

Perthane

Phantolide

phenanthrene

Phenanthrene-1,4-dione

Phenkapton

Phenol

Phenothiazine

Phenothrin I

Phenothrin II

Phenoxyacetic acid

Phenthoate

Phorate

Phorate sulfone

Phorate sulfoxide

Phorate-oxon

Phosalone

Phosfolan

Phosphamidon

Phthalide

Phthalimide

Picloram methyl ester

Picolinafen

Picoxystrobin

Pindone

Piperalin

Piperonyl butoxide

Piperophos

Pirimicarb

Pirimiphos-ethyl

Pirimiphos-methyl

Plifenat

p-Nitrotoluene

Potasan

Prallethrin, cis-

Prallethrin, trans-

Pretilachlor

Probenazole

Prochloraz

Procymidone

Prodiamine

Profenofos

Profenofos metabolite (4-Bromo-2-chlorophenol)

Profluralin

Prohydrojasmon I

Prohydrojasmon II

Promecarb

Promecarb artifact [5-isopropyl-3-methylphenol]

Prometon

Prometryn

Propachlor

Propamocarb

Propanil

Propaphos

Propargite

Propargite metabolite [Cyclohexanol, 2-(4-tert-butylphenoxy)]

Propazine

Propetamphos

Propham

Propiconazole-II

Propisochlor

Propoxur

Propyzamide

Prosulfocarb

Prothioconazole-desthio

Prothiofos

Prothoate

Pyracarbolid

Pyraclofos

Pyraflufen-ethyl

Pyrazon

Pyrazophos

Pyrazoxyfen

pyrene

Pyrethrin I

Pyrethrin II

Pyributicarb

Pyridaben

Pyridaphenthion

Pyridate

Pyridinitril

Pyrifenox I

Pyrifenox II

Pyriftalid

Pyrimethanil

Pyrimidifen

Pyriminobac-methyl (E)

Pyriminobac-methyl (Z)

Pyriproxyfen

Pyroquilon

Quinalphos

Quinoclamine

Quinoxyfen

Quintozene metabolite (pentachlorophenyl methyl sulfide)

Quizalofop-ethyl

Rabenzazole

Resmethrin

Resmethrine II

retene

Rotenone

S,S,S-Tributylphosphorotrithioate

Sebuthylazine

Sebuthylazine-desethyl

Secbumeton

Silafluofen

Silthiopham

Simazine

Simeconazole

Simetryn

Spirodiclofen

Spiromesifen

Spiroxamine I

Spiroxamine II

Spiroxamine metabolite (4-tert-butylcyclohexanone)

Sudan I

Sudan II

Sudan Red

Sulfallate

Sulfanilamide

Sulfentrazone

Sulfotep

Sulfur (S8)

Sulprofos

Swep

Tamoxifen

TCEP

TCMTB

TCPP

Tebuconazole

Tebufenpyrad

Tebupirimifos

Tebutam

Tebuthiuron

Tecnazene

Tefluthrin, cis-

Temephos

Terbacil

Terbucarb

Terbufos

Terbufos-oxon-sulfone

Terbufos-sulfone

Terbumeton

Terbuthylazine

Terbuthylazine-desethyl

Terbutryn

Terrazole

Tetrabromo-o-chlorotoluene

Tetrabromophthalate diol

Tetrachloroguaiacol

Tetrachlorvinphos

Tetraconazole

Tetradifon

Tetraethyl pyrophosphate

Tetrahydrophthalimide, cis-1,2,3,6-

Tetramethrin I

Tetramethrin II

Tetrapropyl thiodiphosphate

Tetrasul

Thenylchlor

Theobromine

Thiabendazole

Thiazopyr

Thifluzamide

Thiofanox

Thiometon

Thionazin

Thymol

Tilt

Tiocarbazil I

Tiocarbazil II

Tolclofos-methyl

Tolfenpyrad

Tolylfluanid

Tolylfluanid metabolite (DMST)

Tolyltriazole [1H-Benzotriazole, 4-methyl-]

Tolyltriazole [1H-Benzotriazole, 5-methyl-]

Tonalide

Toxaphene Parlar 26

Toxaphene Parlar 50

Toxaphene Parlar 62

TPP

Transfluthrin

trans-Nonachlor

Traseolide

Triadimefon

Triadimenol

Triallate

Triamiphos

Triapenthenol

Triazamate

Triazophos

Tribromoneopentyl alcohol

Tributyl phosphate

Trichlamide

Trichlorfon

Trichloronate

Trichlorosyringol

Triclopyr methyl ester

Triclosan

Triclosan-methyl

Tricresylphosphate, meta-

Tricresylphosphate, ortho-

Tricyclazole

Tridemorph , 4-tridecyl-

Tridiphane

Trietazine

Triethyl phosphate

Trifenmorph

Trifloxystrobin

Triflumizole

Trifluralin

triphenylene

Tri-p-tolyl phosphate

Tris(2-butoxyethyl) phosphate

Tris(2-ethylhexyl) phosphate

Triticonazole

Tryclopyrbutoxyethyl

Tycor (SMY 1500)

Uniconizole-P

Vamidothion

Vernolate

Vinclozolin

XMC (3,4-Dimethylphenyl N-methylcarbamate)

XMC (3,5-Dimethylphenyl N-methylcarbamate)

Zinc diethyldithiocarbamate

Zoxamide

Zoxamide decomposition product
