# Supplementary material for: Multi-class chemical exposure in rural Peru using silicone wristbands
Source: J Expo Sci Environ Epidemiol. 2017 Jul 26;27(6):560–8. doi: 10.1038/jes.2017.12 (PMC5658680; doi:10.1038/jes.2017.12)
Supplement: Supplementary Table S2 [file jes201712x2.pdf]

Table S2. Chemicals detected in silicone wristbands with GC-MS screen and summarized by demographic.

				community				gender		occupation		age				
Class	Compound	CASRN[a]	Total # detections	Moyobamba	Rioja	Tingana	Yantalo	Female	Male	Farm Worker	Other	0 - 13	13 - 28	28 - 43	43 - 58	58 - 73
Plasticizer	bis (2-ethylhexyl) phthalate	117-81-7	68	15	15	13	25	35	33	25	43	9	16	14	19	10
	butyl benzyl phthalate	85-68-7	39	9	5	9	16	19	20	15	24	4	9	9	11	6
	dicyclohexyl phthalate	84-61-7	54	12	12	10	20	27	27	21	33	9	13	10	14	8
	diethyl phthalate	84-66-2	64	15	14	13	22	33	31	23	41	8	16	13	17	10
	diisobutyl phthalate	84-69-5	65	14	15	13	23	32	33	25	40	9	13	14	19	10
	dimethyl phthalate	131-11-3	2	---	---	---	2	1	1	1	1	---	---	1	---	1
	di-n-butyl phthalate	84-74-2	68	15	15	13	25	35	33	25	43	9	16	14	19	10
	di-n-hexyl phthalate	84-75-3	4	1	---	1	2	2	2	1	3	2	---	---	1	1
	di-n-nonyl phthalate	84-76-4	15	3	3	5	4	4	11	8	7	2	4	3	5	1
	di-n-octyl phthalate	117-84-0	3	3	---	---	---	1	2	---	3	1	2	---	---	---
	di-n-propyl phthalate	131-16-8	2	1	---	---	1	2	---	---	2	1	---	---	1	---
Industrial	2,4-dichlorophenol	120-83-2	4	1	1	1	1	2	2	2	2	---	1	2	---	1
	3,5-dichloroaniline	626-43-7	4	1	---	---	3	3	1	---	4	1	2	1	---	---
	3-chloro-4-fluoroaniline	367-21-5	1	---	---	---	1	1	---	---	1	---	1	---	---	---
	3-chloroaniline	108-42-9	8	3	1	2	2	5	3	3	5	1	2	4	---	1
	4-chlorophenol	106-48-9	6	1	2	2	1	2	4	3	3	1	3	---	2	---
	4H-cyclopenta[def]phenanthren-4-one	5737-13-3	1	---	1	---	---	1	---	1	---	---	1	---	---	---
	4-methylphenol	106-44-5	11	---	4	4	3	2	9	9	2	---	3	---	4	4
	9-fluorenone	486-25-9	7	---	1	2	4	5	2	3	4	---	2	2	2	1
	biphenyl	92-52-4	4	---	1	1	2	3	1	1	3	---	1	1	1	1
	dibenzofuran	132-64-9	4	---	1	2	1	3	1	2	2	---	---	1	2	1
	diphenylamine	122-39-4	1	---	1	---	---	1	---	---	1	1	---	---	---	---
	sulfur (S_8)	10544-50-0	1	---	---	---	1	---	1	---	1	---	1	---	---	---
	m -cresol	108-39-4	1	---	---	1	---	---	1	1	---	---	---	---	1	---
Flame retardant	tributyl phosphate	126-73-8	1	---	---	---	1	1	---	---	1	---	---	---	---	1
	triphenyl phosphate	115-86-6	38	10	6	4	18	22	16	8	30	6	8	8	10	6
	tris (2-butoxyethyl) phosphate	78-51-3	1	1	---	---	---	---	1	---	1	---	1	---	---	---
	tris (2-ethylhexyl) phosphate	78-42-2	1	---	---	---	1	---	1	---	1	---	---	---	1	---
	tris (3-chloropropyl) phosphate	26248-87-3	5	2	---	1	2	2	3	1	4	2	---	---	2	1
PCPP	benzophenone	119-61-9	31	12	3	3	13	20	11	5	26	8	9	5	8	1
	benzyl benzoate	120-51-4	18	7	1	2	8	11	7	4	14	2	3	5	6	2
	butylated hydroxyanisole	25013-16-5	16	7	2	1	6	14	2	2	14	4	3	4	4	1
	caffeine	58-08-2	38	10	7	7	14	19	19	13	25	4	8	6	14	6
	cashmeran	33704-61-9	2	---	1	---	1	2	---	1	1	1	---	1	---	---
	celestolide	13171-00-1	2	1	---	---	1	2	---	---	2	---	---	2	---	---
	eugenol	97-53-0	20	5	4	2	9	16	4	6	14	2	3	3	6	6
	galaxolide	1222-05-5	66	15	15	13	23	35	31	23	43	9	16	14	18	9
	musk amberette	83-66-9	1	---	---	---	1	---	1	1	---	---	---	---	---	1
	musk ketone	81-14-1	21	11	1	1	8	17	4	2	19	2	7	7	3	2
	nicotine	54-11-5	5	1	1	3	---	2	3	4	1	1	---	---	4	---
	thymol	89-83-8	6	---	2	---	4	4	2	1	5	1	2	2	1	---
	tonalide	1506-02-1	60	15	8	13	24	33	27	20	40	9	14	13	17	7
	traseolide	68140-48-7	1	1	---	---	---	1	---	---	1	---	1	---	---	---
	triclosan	3380-34-5	16	6	1	5	4	6	10	6	10	3	4	3	4	2
PAH	1,2-dimethylnaphthalene	573-98-8	2	---	1	---	1	1	1	1	1	---	1	---	1	---
	1,6-dimethylnaphthalene	575-43-9	1	---	---	---	1	1	---	---	1	---	1	---	---	---
	1-methylnaphthalene	90-12-0	9	---	3	1	5	5	4	4	5	1	2	1	2	3
	1-methylphenanthrene	832-69-9	25	---	8	5	12	12	13	12	13	3	8	3	8	3
	1-methylpyrene	2381-21-7	2	---	2	---	---	1	1	1	1	1	---	---	1	---
	2,6-diethylnaphthalene	59919-41-4	5	---	2	---	3	2	3	1	4	2	1	---	1	1
	2,6-dimethylnaphthalene	581-42-0	3	---	1	---	2	1	2	2	1	---	1	---	1	1
	2-ethylnaphthalene	939-27-5	2	---	1	---	1	1	1	1	1	---	1	---	1	---
	2-methylanthracene	613-12-7	6	---	2	1	3	4	2	3	3	---	4	---	1	1

Table S1. Chemicals detected in silicone wristbands with GC-MS screen and summarized by demographic.*cont.*

				community				gender		occupation		age				
Class	Compound	CASRN[a]	Total # detections	Moyobamba	Rioja	Tingana	Yantalo	Female	Male	Farm Worker	Other	0 - 13	13 - 28	28 - 43	43 - 58	58 - 73
	2-methylnaphthalene	91-57-6	3	---	2	---	1	1	2	1	2	---	1	---	---	2
	2-methylphenanthrene	2531-84-2	22	---	8	2	12	11	11	10	12	4	5	4	6	3
	3,6-dimethylphenanthrene	1576-67-6	7	---	4	---	3	2	5	3	4	1	1	---	4	1
	9-methylanthracene	779-02-2	1	---	---	---	1	1	---	---	1	---	1	---	---	---
	acenaphthylene	208-96-8	13	---	1	3	9	11	2	4	9	2	4	2	3	2
	anthracene	120-12-7	46	10	8	10	18	26	20	16	30	8	12	8	13	5
	benzo[a]anthracene	56-55-3	7	---	3	1	3	6	1	2	5	1	3	1	1	1
	benzo[a]fluorene	238-84-6	2	---	1	1	---	1	1	2	---	---	2	---	---	---
	benzo[b]fluorene	243-17-4	2	---	1	---	1	2	---	1	1	---	2	---	---	---
	benzo[e]pyrene	192-97-2	1	---	1	---	---	1	---	1	---	---	1	---	---	---
	chrysene	218-01-9	1	---	1	---	---	1	---	1	---	---	1	---	---	---
	cyclopenta(cd)pyrene	27208-37-3	2	---	2	---	---	2	---	1	1	---	1	---	---	1
	dibenzothiophene	132-65-0	1	---	1	---	---	---	1	1	---	---	---	---	1	---
	fluoranthene	206-44-0	40	5	10	10	15	21	19	16	24	8	8	8	10	6
	fluorene	86-73-7	16	1	5	2	8	10	6	6	10	1	6	3	2	4
	naphthalene	91-20-3	16	6	2	---	8	13	3	1	15	5	4	2	1	4
	phenanthrene	85-01-8	27	2	9	7	9	15	12	14	13	2	6	8	6	5
	pyrene	129-00-0	30	4	6	7	13	17	13	12	18	5	9	3	9	4
	retene	483-65-8	4	---	3	---	1	2	2	1	3	1	---	---	2	1
	triphenylene	217-59-4	1	---	1	---	---	1	---	---	1	---	---	---	---	1
Insecticide	promecarb artifact	3228-03-3	5	---	1	2	2	2	3	2	3	1	2	1	1	---
	bendiocarb	22781-23-3	1	---	---	---	1	---	1	1	---	---	---	---	---	1
	benzoximate metabolite	55440-55-6	1	---	---	---	1	1	---	---	1	---	---	---	1	---
	bioallethrin	584-79-2	1	---	1	---	---	1	---	---	1	---	---	1	---	---
	chlorpyrifos	2921-88-2	18	---	8	3	7	8	10	10	8	2	2	3	6	5
	cyhalothrin (gamma)	76703-62-3	1	---	---	---	1	---	1	1	---	---	---	---	1	---
	cyhalothrin (lambda)	68085-85-8	1	---	---	---	1	---	1	1	---	---	---	---	1	---
	cypermethrin I	52315-07-8	1	---	1	---	---	---	1	1	---	---	---	---	---	1
	cypermethrin II	52315-07-8	3	1	1	---	1	---	3	1	2	---	---	1	1	1
	cypermethrin III	52315-07-8	1	1	---	---	---	1	---	---	1	---	1	---	---	---
	cyphenothrin-cis	39515-40-7	2	---	---	---	2	---	2	1	1	1	---	---	---	1
	cyphenothrin-trans	39515-40-7	1	---	---	---	1	---	1	---	1	1	---	---	---	---
	dichlorvos	62-73-7	2	---	---	---	2	2	---	---	2	---	2	---	---	---
	dimethoate	60-51-5	1	---	---	---	1	---	1	---	1	---	---	---	1	---
	fipronil	120068-37-3	1	---	---	---	1	1	---	---	1	---	---	---	---	1
	malathion	121-75-5	1	---	---	---	1	---	1	1	---	---	---	---	---	1
	methamidophos	10265-92-6	1	---	---	1	---	---	1	1	---	---	---	1	---	---
	N,N'-diethyl-*m*-toluamide	134-62-3	30	5	2	10	13	13	17	15	15	2	7	6	9	6
	p,*p*'-DDD	72-54-8	10	1	1	4	4	3	7	6	4	1	---	1	4	4
	p,*p*'-DDE	72-55-9	14	3	1	5	5	3	11	7	7	1	---	3	6	4
	p,*p*'-DDT	50-29-3	14	---	---	6	8	4	10	9	5	1	1	1	6	5
	permethrin-cis	52645-53-1	2	---	---	---	2	2	---	---	2	---	1	---	---	1
	permethrin-trans	61949-77-7	1	---	---	---	1	1	---	---	1	---	---	---	---	1
	piperonyl butoxide	51-03-6	9	---	---	3	6	3	6	5	4	1	1	1	4	2
	pirimiphos-methyl	29232-93-7	3	3	---	---	---	2	1	---	3	1	---	2	---	---
	promecarb	2631-37-0	5	3	---	---	2	4	1	1	4	---	2	2	1	---
Herbicide	diuron metabolite	102-36-3	13	4	2	1	6	6	7	4	9	2	5	4	---	2
	butachlor	23184-66-9	12	---	9	---	3	6	6	4	8	2	4	2	2	2
	difenoxuron	14214-32-5	1	1	---	---	---	1	---	---	1	---	---	---	---	1
	terbutryn	886-50-0	1	---	---	---	1	1	---	---	1	---	1	---	---	---
Fungicide	cyprodinil	121552-61-2	1	1	---	---	---	---	1	---	1	1	---	---	---	---
	tebuconazole	107534-96-3	7	---	7	---	---	3	4	3	4	1	3	2	1	---
	trifloxystrobin	141517-21-7	9	---	8	---	1	4	5	4	5	1	3	3	2	---

[a] CASRN was not always available for individual isomers.
